# Recent Advances in Integrated Photonic Sensors

**DOI:** 10.3390/s121115558

**Published:** 2012-11-09

**Authors:** Vittorio M. N. Passaro, Corrado de Tullio, Benedetto Troia, Mario La Notte, Giovanni Giannoccaro, Francesco De Leonardis

**Affiliations:** Photonics Research Group, Dipartimento di Elettrotecnica ed Elettronica, Politecnico di Bari, Via Edoardo Orabona 4, 70125 Bari, Italy; E-Mails: corrado.detullio@yahoo.com (C.T.); b.troia@poliba.it (B.T.); lanottemario@alice.it (M.L.N.); g.giannoccaro@poliba.it (G.G.); f.deleonardis@poliba.it (F.D.L.)

**Keywords:** chemical sensors, Fano resonance, Mach-Zehnder interferometer, Raman effect, ring resonator, silicon-on-insulator

## Abstract

Nowadays, optical devices and circuits are becoming fundamental components in several application fields such as medicine, biotechnology, automotive, aerospace, food quality control, chemistry, to name a few. In this context, we propose a complete review on integrated photonic sensors, with specific attention to materials, technologies, architectures and optical sensing principles. To this aim, sensing principles commonly used in optical detection are presented, focusing on sensor performance features such as sensitivity, selectivity and rangeability. Since photonic sensors provide substantial benefits regarding compatibility with CMOS technology and integration on chips characterized by micrometric footprints, design and optimization strategies of photonic devices are widely discussed for sensing applications. In addition, several numerical methods employed in photonic circuits and devices, simulations and design are presented, focusing on their advantages and drawbacks. Finally, recent developments in the field of photonic sensing are reviewed, considering advanced photonic sensor architectures based on linear and non-linear optical effects and to be employed in chemical/biochemical sensing, angular velocity and electric field detection.

## 1. Introduction to Integrated Photonic Sensors

Waveguide-based devices are becoming more and more attractive in the field of optical elaboration of signals for sensing applications in different areas, especially in chemical and bio-chemical detection, angular rate rotation estimation and electric field detection [1–3]. The interest in optical sensing is justified by incomparable advantages enabled by photonic technologies, such as high sensitivity, possibility of integration with electronic devices, compactness, metal-free operation, low-cost and electromagnetic immunity. The significant performance of integrated photonic sensors is allowed by technological features and developments, among which ring resonators and surface plasmons have attracted the interest of researchers over the last few years [4, 5]. In the following sections, the most attractive characteristics of these technological improvements in the field of optical sensing are considered.

Nowadays, biochemical sensing based on ring resonators (RRs) is a very intriguing technological platform. In fact, the possibility of employing optical principles and effects similar to those commonly adopted in traditional straight waveguides, allows ultra-high sensing performance and efficient and CMOS-compatible readout schemes to be achieved. Moreover, it is possible to improve sensing performance because of the optical field enhancement in small areas, the high quality factor *Q* and the small dimensions characterizing overall sensor architectures [6]. In case of chemical and biochemical sensing, the operating principle consists of the variation of the effective index of the optical mode propagating into the structure, as a consequence of the presence of a chemical substance to be detected close to the sensor surface. An optimal design of RR-based devices can be obtained by considering figures of merit related to different types of resonators, such as planar RRs or liquid core optical RRs. Moreover, sensor performance can be analyzed as a function of geometric and optical parameters, such as waveguide sizes, ring radius and operating wavelength. RR-based sensors can be also employed for the realization of integrated optical gyroscopes (IOGs) for estimating the angular velocity in inertial systems. In this case, the operating principle characterizing the photonic sensing mechanism is the Sagnac effect, which leads to a phase shift between two counter-propagating beams proportional to the angular velocity at which the device is rotating [7]. Different configurations have been proposed for these devices, with characteristics suitable for different applications [8]. Unlike fiber optical gyroscopes (FOG), which are based on the same operating principle, IOGs present the fundamental advantages of lower dimensions and CMOS technology integration. Moreover, performance of IOGs are comparable with those of other technologies, such as mechanical, vibrational and MEMS gyros [9, 10]. Innovative photonic sensors of electric field are also realized with structures based on straight waveguides as well as on RRs [11].

Surface Plasmon Resonance (SPR) is an intriguing technique commonly employed in integrated photonic sensors designed for the detection of chemical and biological species, some of which have been reviewed in [12]. Two different basic schemes have been proposed in order to realize optical sensing into planar waveguides based on surface plasmon polaritons (SPP). The first scheme requires the excitation of a surface plasmon wave, while the other one consists in the excitation of “pure” plasmons [13]. In any case, the sensing principle is always based on the localized variation of the refractive index near the waveguide surface, resulting in the modification of an excited surface plasmon wave or SPP at the dielectric-metal interface.

## 2. Optical Sensing Principles

In this section, the main effects employed in optical sensing detection schemes are presented, focusing on the abovementioned fields of application. In particular, it is convenient to distinguish among three different sensing mechanisms usually employed in label-free optical detection: homogeneous sensing, surface sensing and optical absorption.

In addition, integrated optical sensors designed for the quantification of angular velocity in gyro systems are based on Sagnac effect, exploited in fiber gyroscopes, too. Another fundamental principle of operation is based on the well-known Fano resonance, employed in electrical field optical sensing.

### 2.1. Homogeneous, Surface and Absorption-Based Sensing

Optical sensing of chemical species is typically based on the variation of waveguide optical properties in the presence of some target analyte near the sensor surface. In particular, the principal sensing mechanisms exploited for this specific application include the variation of waveguide effective index or waveguide absorption coefficient, as a function of the concentration of the chemical species to be sensed.

**Figure 1.**
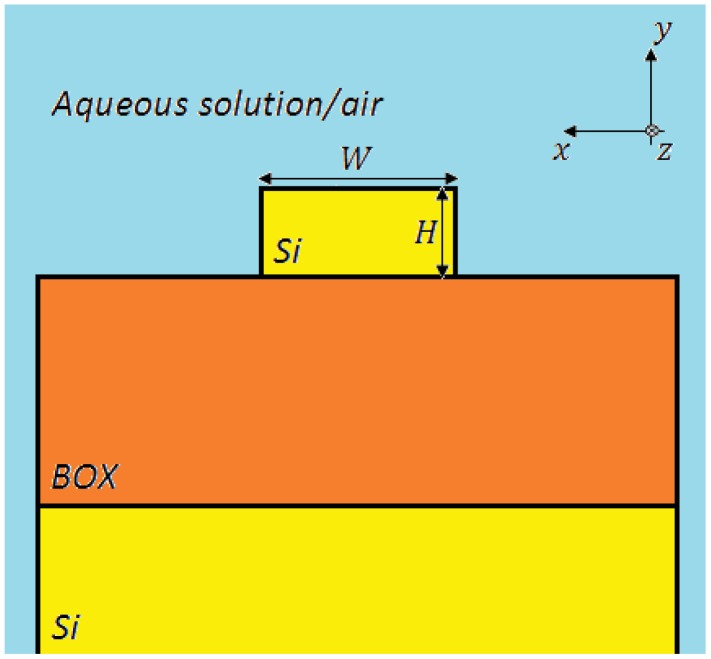
Example of a SOI photonic wire waveguide based bio-sensor.

Optical waveguides realized in silicon on insulator (SOI) technology are typically composed of a top silicon layer, a few hundreds of nanometers thick, a 1–2 μm-thick buried oxide (BOX) substrate, and a bottom silicon layer (several hundreds of μm thick), which is a support for the optical chip. The main advantage consists in a large refractive index contrast (∼2 at *λ* = 1.55 μm), since the refractive indices of silicon and BOX are equal to *n_Si_* = 3.476 and *n_Ox_* = 1.444 at *λ* = 1.55 μm, respectively. The cover medium (cladding) could be an aqueous solution, in which the analyte is dissolved (Figure 1), or simply air, depending on the sensing application.

The principle of operation of optical guided-wave bio-chemical sensors in case of homogeneous and surface sensing can be described as follows [14]. When light propagates into an optical waveguide, a certain amount of power travels into the core, while the remainder is confined into the cladding and substrate regions (*i.e.*, SiO_2_). The effective index of the propagating optical field also depends on the concentration of the specific analyte or gas localized in the cover medium, near the sensor surface. Consequently, the magnitude of the effective index change is related to the percentage of field interacting with the analyte, and thus to the confinement factor in the medium where the analyte is concentrated. Various gases and chemical species can be detected exploiting homogeneous sensing. For example, in case of glucose or ethanol detection, the optical waveguide is covered by an aqueous solution (*n_c_* = 1.33 at *λ* = 1.55 μm), in which the analyte has to be dissolved. The dimensionless waveguide sensitivity, *S_h_*, can be evaluated as follows [14–16]:

(1) $$S_{h}={\frac{\partial n_{\text{eff}}}{\partial n_{c}}|}_{n_{c}=n_{c}^{0}}=\frac{2n_{c}^{0}}{Z_{0}P}\iint_{C}{\left|\vec{E}(x,y)\right|}^{2}\text{dxdy}=\frac{2n_{c}^{0}\iint_{\infty }{\left|\vec{E}(x,y)\right|}^{2}\text{dxdy}}{Z_{0}P}\Gamma_{C}^{I}$$

where:

(2) $$P=\iint_{\infty }[(\bar{E}\times {\bar{H}}^{*}+{\bar{E}}^{*}\times \bar{H})\cdot \bar{z}]\text{dxdy}$$

In Equation (1), *Z*_0_ is the free space impedance, *n_eff_* is the effective mode index, *n_c_* is the solution refractive index, $n_{c}^{0}$ is the aqueous solution refractive index in absence of the analyte, *Ē* and *H̄* are the electric and magnetic field vectors, respectively, and $\Gamma_{C}^{I}$ is the optical field intensity confinement factor in the cladding region, defined as follows [14]:

(3) $$\Gamma_{C}^{I}=\frac{\iint_{C}{\left|\vec{E}(x,y)\right|}^{2}\text{dxdy}}{\iint_{\infty }{\left|\vec{E}(x,y)\right|}^{2}\text{dxdy}}$$

The integration domain indices, *i.e.*, *C* and *∞*, stands for cladding cross section and whole computational domain, respectively. Unlike homogeneous sensing, surface sensing is based on the selective immobilization of receptor molecules on the functionalized waveguide surface. These molecules form a very thin adlayer on the waveguide surface. Consequently, the increase of the molecular adsorbed layer (adlayer) thickness causes the effective index change Δ*n_eff_*. According with the variational theorem, the effective mode index will change as [14]:

(4) $$\Delta n_{\text{eff}}=\frac{n_{m}^{2}-{\left(n_{C}^{0}\right)}^{2}}{Z_{0}P}\iint_{\Sigma }{\left|\vec{E}(x,y)\right|}^{2}\text{dxdy}$$

where *n_m_* is the refractive index of the molecular adlayer and *Σ* represents the region in which the adlayer increases. Similarly to the definition of sensitivity given for homogeneous sensing, it is possible to define the surface waveguide sensitivity as follows [14]:

(5) $$S_{s}={\frac{\partial n_{\text{eff}}}{\partial \rho }|}_{n_{c}=n_{c}^{0}}$$

where *ρ* is the thickness of the molecular adlayer [17–20].

Another operating principle to be exploited in photonic chemical sensors is the optical absorption. In particular, the absorption coefficient of the waveguide *α*, which is a function of the operative wavelength, electronic and photonic properties of the material, is not the only cause of optical absorption. In fact, several gases, organic and inorganic molecules are characterized by specific absorption spectra in the near- and mid-infrared (IR) wavelength regions. For example, methane (CH_4_), carbon dioxide (CO_2_) or sulfur dioxide (SO_2_) present an unique absorption spectrum in the mid-IR. In particular, the frequency position of vibrational and rotational-vibrational transitions can provide information about the chemical composition of a monitored molecule.

This principle is useful for optical sensing, since it is possible to connect the optical signal intensity to the gas or analyte concentration *C* through the Beer-Lambert law [21]:

(6) $$\begin{array}{cc} I=I_{0}\;\text{exp}(-\alpha L), & \alpha =C\varepsilon \end{array}$$

where *I* and *I*_0_ are the light intensities at the end and at the beginning of waveguide path length, respectively, *L* is the optical path and *ε* represents the molar absorption coefficient of the analyte. Sensing mechanism consists in monitoring the optical signal intensity changes as a function of the absorption spectrum of the chemical specie to be detected. In particular, as shown in Equation (6), the absorption coefficient *α* is linearly dependent on *I*_0_ and *C*. Consequently, at the output of the waveguide it is possible to register sharp peaks in the transmission spectra at specific wavelengths corresponding to the molecule absorption lines.

### 2.2. Sagnac Effect

**Figure 2.**
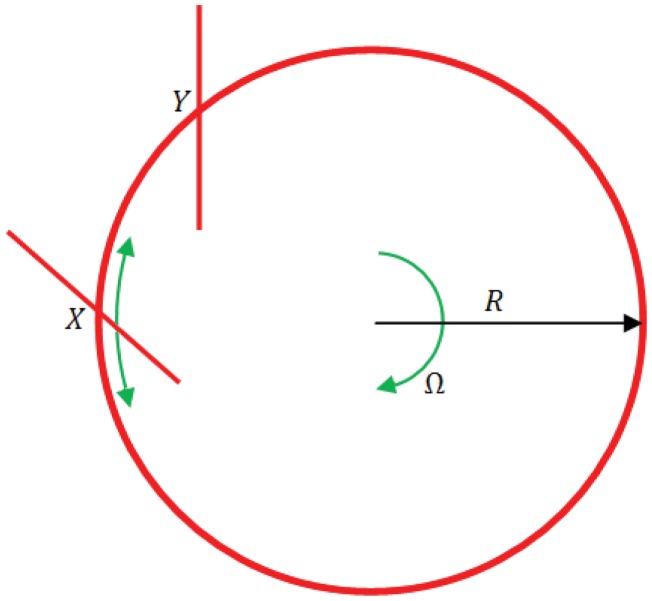
Circular rotating (Sagnac) interferometer.

The Sagnac effect has been widely employed in fiber optic based sensor for pressure, temperature and torsion monitoring [22–24]. Moreover, it is the most exploited operating principle in optical gyroscopes [25]. The detection of the angular velocity relies upon different effective path lengths experienced by two counter-propagating beams of light in a closed path. The following calculation demonstrates how it is possible to relate the effective path length to turn rate about an axis perpendicular to the plane containing the light path [26]. By considering light propagating around the circumference of a circle of radius R into a perfect stationary circular interferometer (Figure 2), if light is coupled into the ‘ring’ and two counter-propagating beams are generated by a beam splitter positioned at the point X, the two beams will recombine each other at the same point.

When the ring is stationary, the time for the light to make one complete round trip around the ring is identical for both beams and is given by:

(7) $$t=\frac{2\pi R}{c}$$

where *c* is the velocity of light which is considered to be invariant, and *t* is called transit time. However, when the interferometer is rotated with angular velocity Ω, each light beam employs a different time to pass around the circumference, since the beam splitter rotates while the light propagates into the ring.

Figure 2 shows that the beam splitter will move to position Y, so that light travelling in a clockwise direction will take a higher path with respect to the condition in which the ring is not rotating. The situation is opposite for the counter-clockwise beam. In general, it is possible to state that light travelling with the direction of rotation must travel further with respect to the case in which the interferometer is stationary, while light travelling against the direction of rotation will have its path length reduced if compared with the stationary condition. Hence, the single pass transit time for the two beams is given by:

(8) $$\begin{array}{cc} \text{Clockwise path} & t_{1}=\frac{2\pi R+\Delta L_{+}}{c} \end{array}$$

(9) $$\begin{array}{cc} \text{Counter}-\text{clockwise path} & t_{2}=\frac{2\pi R-\Delta L_{-}}{c} \end{array}$$

where *ΔL*_+_ = *RΩt*_1_ and *ΔL*_−_ = *RΩt*_2_ are the increment and decrement in the path length, respectively. This can also be interpreted as the velocity of light is different for the two counter-propagating beams travelling along the same path length. The difference in transit time, *Δt*, is given from the difference between *t_1_* and *t_2_*, as follows:

(10) $$\Delta t=t_{1}-t_{2}=2\pi R\left(\frac{1}{c-R\Omega }-\frac{1}{c+R\Omega }\right)$$

Consequently, this becomes:

(11) $$\Delta t=\frac{4\pi R^{2}\Omega }{c^{2}}$$

The optical path length difference can be expressed as:

(12) $$\Delta L=c\Delta t=\frac{4\pi R^{2}\Omega }{c}$$

The area *A* enclosed by the path length is πR^2^. Hence Equation (12) may be rewritten in the form:

(13) $$\Delta L=\frac{4A\Omega }{c}$$

From this discussion it is possible to understand that the optical path difference *ΔL* does not depend on the position of the rotation axis. Hence, measurement of the optical path difference enables an observer, located on a rotating reference frame, to measure the so-called absolute rotation of his reference frame.

### 2.3. Raman Effect

The Raman effect is an inelastic scattering process which is becoming widely used in photonic devices, especially in those fabricated using optical fibers or SOI technology. The most promising features of Raman scattering in silicon are:

- The Raman gain coefficient is more than 100 times larger than that of silica glass in the telecommunication band;
- The high refractive index contrast between silicon and silica leads to strong confinement of optical modes inside silicon-on-insulator (SOI) waveguides and enables the achievement of large intensities at moderate input optical powers.

However, modeling and study of photonic sensors in SOI technology have to include other non-linear effects which are detrimental for the performance of the device. Among these processes, the most relevant are free carrier absorption (FCA) and two photon absorption (TPA), which take place in silicon waveguides at telecommunication wavelengths [27, 28].

The Raman effect arises from fluctuations in the optical properties of a medium. It is important to distinguish among three different scattering effects in optical fibers:

- Rayleigh scattering;
- Brillouin scattering;
- Raman scattering.

Rayleigh scattering is an elastic process since the energies of incident and scattered photons are the same. On the contrary, Raman and Brillouin scattering are inelastic effects. In fact, incident and scattered photons have different energies and the energy lost by the incident field leads to creation of phonons, which modify the vibrational states of the medium. The major difference between the Brillouin and Raman scattering effects concerns the different frequencies of the scattered phonons, since the former involves low frequency phonons, called acoustic ones, and the latter involves phonons at high frequency, called optical phonons.

The nature of Raman scattering leads to two different forms of this phenomenon, that are spontaneous Raman scattering and stimulated Raman scattering (SRS). Spontaneous Raman scattering arises when interaction between light and molecules of the medium is mainly excited by thermal effects. By considering an incident wave at frequency υ*_i_*, the interaction leads to the generation of a phonon at frequency Ω, and so to the generation of a scattered wave at a frequency equal to *υ_o_* = *υ*_i_ − Ω. Interaction can also lead to annihilation of a phonon, so that the scattered light is at frequency *υ_o_* = *υ*_i_ + Ω. Down shifted and up shifted frequency waves are called Stokes and anti-Stokes, respectively. The frequency Ω of phonons involved in Raman scattering is related to the normal vibration mode of the medium, so that properties of scattered light can be related to the physical characteristics of the medium.

The occurrence of stimulated Raman scattering can be understood by considering that phonons can be created through a scattering process or through a thermal mechanism. In the case of low intensities of the incident light, according to thermal equilibrium the two processes are balanced, so that the density of phonons is constant and the optical properties of the medium do not change. Instead, when the intensity of light is larger than a certain threshold value, variations of the optical properties of the medium lead to an enhancement of scattering process by several orders of magnitude, and photon emission can be stimulated by the presence of another photon, leading to optical gain.

In reality, both spontaneous and stimulated emission simultaneously occurs. If stimulated emission is triggered by a photon that is part of a signal, we are in the presence of optical gain, while if emission is triggered by a photon generated by spontaneous emission, we are in the presence of an amplified spontaneous emission noise. In case of photonic sensors, stimulated emission is a fundamental effect since it can give rise to optical amplification and lasing, while spontaneous emission is generally a detrimental effect since it leads to excess noise in the system [29, 30].

**Figure 3.**
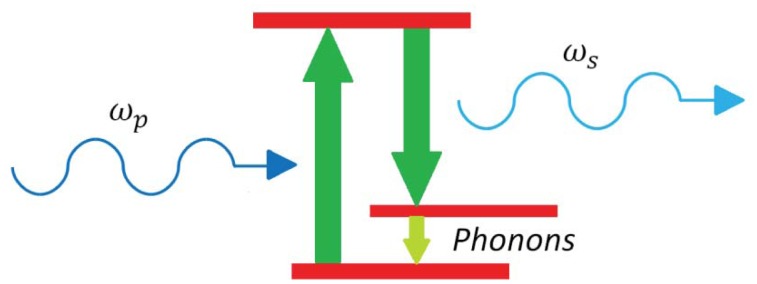
Schematic of Stimulated Raman Scattering.

By modeling SRS in continuous wave (CW) and quasi-CW conditions and considering propagation of only pump and Stokes waves, the variation of the Stokes wave during the propagation can be described by [29]:

(14) $$\frac{dI_{s}}{dz}=g_{R}I_{p}I_{s}$$

where *I_s_* and *I_p_* are the Stokes and pump intensities, respectively, and *g_R_* is the Raman gain coefficient [31]. This last term is related to the imaginary part of the third-order nonlinear susceptibility through the relation:

(15) $$g_{R}=\frac{4\omega_{s}\chi_{R}}{cn}$$

where *ω_s_* is the angular frequency of the Stokes wave, *χ_R_* is the Raman resonant susceptibility and *n* is the refractive index of the medium. The Raman resonant susceptibility can be expressed as [29]:

(16) $$\chi_{R}(\omega_{s},\Delta \omega =\omega_{p}-\omega_{s},\omega_{p})=\frac{2\Omega_{R}\Gamma_{R}\xi_{R}}{2j\Gamma_{R}\Delta \omega +\Omega_{R}^{2}-\Delta \omega^{2}}$$

where *ω_s_* and *ω_p_* are the Stokes and pump angular frequencies, respectively, Ω_R_ is the Raman shift at which the gain is maximum, ξ_R_ is the Raman susceptibility when Δω = Ω_R_, Γ_R_ is the resonance half width. Equation (14) is useful for understanding the dependence of the Raman gain coefficient on the Raman shift. Silicon presents a narrow Raman-gain spectrum being Ω_R_/2*π* = 15.6 THz. A schematic of SRS is reported in Figure 3.

Stimulated Raman scattering can be studied through a system of coupled differential equations. By considering only the effect of absorption for pump and Stokes wave and *P_s_* = *I_s_A_eff_*, it is possible to write [29]:

(17) $$\frac{\partial P_{s}}{\partial z}=\frac{g_{R}}{A_{\text{eff}}}P_{p}P_{s}-\alpha_{s}P_{s}$$

(18) $$\eta \frac{\partial P_{p}}{\partial z}=\frac{\omega_{p}}{\omega_{s}}\frac{g_{R}}{A_{\text{eff}}}P_{p}P_{s}-\alpha_{p}P_{p}$$

where *P_s_* and *P_p_* are Stokes and pump power, *α_s_* and *α_p_* are the losses at Stokes and pump frequencies, respectively, and *η* = ±1 depending on the fact that signals propagate in the same or opposite directions.

The Raman effect can be employed for sensing purposes. In particular, the non-linear surface-enhanced Raman scattering (*i.e.*, SERS, a sensitive technique able to detect the enhancement in Raman scattering when target molecules are adsorbed onto a metal surface), represents a spectroscopic tool for non-invasive and non-destructive detection of biochemical and chemical molecules. In this context, novel nanocone structures have been proposed for the detection of single amino acid phosphorylation [32]. Moreover, the use of SERS in conventional resonant microcavities has been well investigated by White *et al.* [33]. In particular, a lab-on-a-chip based on liquid core optical ring resonator (LCORR) has been proposed for the detection of Rhodamine 6G (R6G) in silver colloid, revealing a detection limit as low as 400 pmol/L. The non-linear Raman effect has been also employed in integrated SOI microcavities (*i.e.*, racetrack resonators) for sensing purposes (e.g., gas detection in mid infrared, angular velocity estimation in gyroscopes) [29, 34]. Several details will be described in the following sections of the review.

### 2.4. Fano Resonance

**Figure 4.**
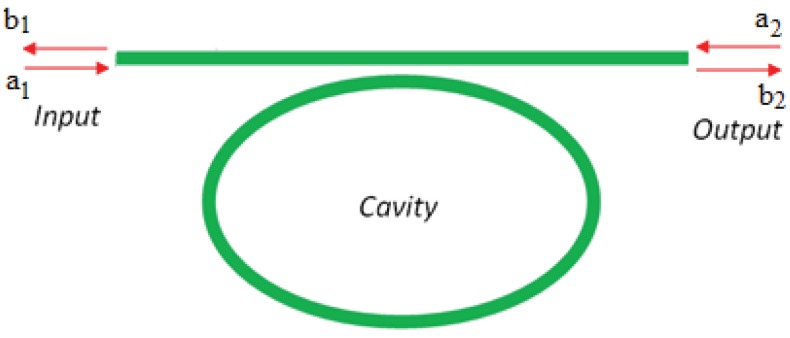
Optical cavity coupled to a straight waveguide.

**Figure 5.**
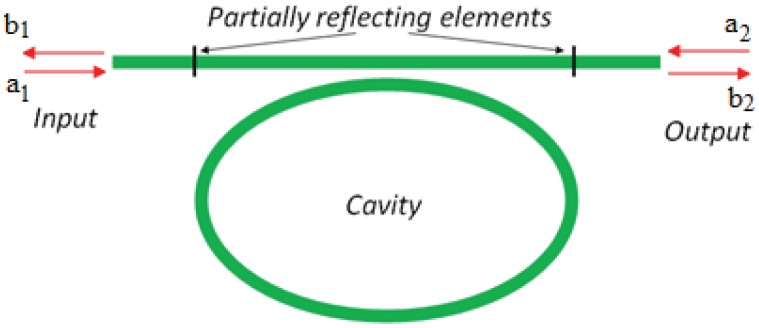
Optical cavity coupled to a straight waveguide with two partially reflecting elements.

Integrated photonic sensors designed for electrical field detection are generally constituted by interferometer architectures, but planar resonant microcavities can be also used. Their sensitivity can be significantly enhanced if the spectral response of the ring resonator presents asymmetric resonances instead of the symmetric typical Lorentzian line-shape of a single cavity [35]. To this purpose, the shape of the resonant spectrum in the transition between 0% and 100% of reflectivity, can be steeper by realizing a Fano resonance with two coupled cavities [36]. In particular, it is possible to obtain an asymmetric transition with a narrower linewidth than that exhibited by a single cavity. Figure 4 shows an optical ring resonator coupled to a straight bus waveguide. In addition, Figure 5 represents a ring resonator coupled to a Fabry-Perot cavity realized on the bus waveguide by inserting two partially reflecting elements, one at the input and the other at the output.

The joint effect of two integrated resonant cavities enables the excitation of Fano resonance. To this end, the effect induced in the configuration sketched in Figure 5 can be explained by using the transfer matrix method.

The transfer matrix of the single-mode cavity coupled to a straight bus waveguide (Figure 4) can be written as follows [37, 38]:

(19) $$\left[\begin{array}{c} b_{2}\\ a_{2} \end{array}\right]=\left[\begin{array}{cc} 1-\frac{i\gamma }{\omega -\omega_{0}} & \frac{-i\gamma }{\omega -\omega_{0}}\\ \frac{i\gamma }{\omega -\omega_{0}} & 1+\frac{i\gamma }{\omega -\omega_{0}} \end{array}\right]\;[\begin{array}{c} a_{1}\\ b_{1} \end{array}]$$

where *a*_1_ and *b*_1_ are the incoming and outcoming amplitudes on one side of the cavity, respectively, while *a*_2_ and *b*_2_ are those on the other side. The center frequency and the width of the cavity resonance are *ω*_0_ and *γ*. In Equation (20), it is possible to calculate the reflection coefficient as:

(20) $$R(\omega )=\frac{\gamma^{2}}{{\left(\omega -\omega_{0}\right)}^{2}+\gamma^{2}}$$

The reflecting elements in the structure shown in Figure 5 modify the phase of the waves transmitted into the waveguide. This effect can be represented by the transfer matrix as in Equation (21):

(21) $$\left[\begin{array}{c} b_{2}\\ a_{2} \end{array}\right]=-\frac{1}{1-r^{2}}\left[\begin{array}{cc} -1 & -r\\ r & 1 \end{array}\right]\;\left[\begin{array}{cc} e^{i\delta } & 0\\ 0 & e^{i\delta } \end{array}\right]\times \left[\begin{array}{cc} 1-\frac{i\gamma }{\omega -\omega_{0}} & \frac{-i\gamma }{\omega -\omega_{0}}\\ \frac{i\gamma }{\omega -\omega_{0}} & 1+\frac{i\gamma }{\omega -\omega_{0}} \end{array}\right]\;\left[\begin{array}{cc} e^{i\delta } & 0\\ 0 & e^{i\delta } \end{array}\right]\;\left[\begin{array}{cc} -1 & -r\\ r & 1 \end{array}\right]\;\left[\begin{array}{c} a_{1}\\ b_{1} \end{array}\right]$$

where *δ* = *ω*/*c* is the phase shift due to the propagation from reflecting element to the cavity and *r* is the reflection coefficient. In this case, the transmissivity can be obtained [36] as:

(22) $$t=\frac{\left(r^{2}-1)e^{2i\delta }(\omega -\omega_{0}\right)}{-e^{4i\delta }r^{2}(\omega -\omega_{0}-i\gamma )-2e^{2i\delta }r^{2}(i\gamma )r+\omega -\omega_{0}+i\gamma }$$

The spectral response of the structure critically depends on the relative position between the resonant frequency and the Fabry-Perot oscillations. In particular, if the resonant frequency corresponds to a maximum in Fabry-Perot oscillations, the structure behaves as a narrow bandwidth reflector with a symmetric Lorentian-like lineshape resonance. If this condition is not satisfied, the resonance spectrum presents an asymmetric sharp variation between 0% and 100% transmission frequencies denoted by *ω_r_* = *ω*_0_ and *ω_t_*, respectively. The frequency shift between these two critical frequencies can be calculated [36] as:

(23) $$\omega_{t}-\omega_{0}=\frac{1+r^{2}-2r\cos(2\delta )}{2r\sin(2\delta )}\gamma$$

where it is possible to consider the phase shift as a constant in the resonance bandwidth and, consequently, *δ*(*ω*) = *δ*(*ω* = *ω*_0_). It is possible to note from Equations (20) and (23) that the two coupled cavities allow a specific bandwith with a lower phase shift to be achieved. Moreover, the fact that the asymmetric shape is obtainable for a wide range of parameters allows one to obtain the desired resonance condition even in the presence of technological imperfections. Finally, ultra-high sensitivities of the order of ∼10^−8^ RIU have been experimentally demonstrated in silicon microring resonators integrated in a Fabry-Perot resonant cavity [39].

## 3. Photonic Sensors for Chemical and Biochemical Detection

Recent developments in photonic technologies lead to the investigation and fabrication of photonic integrated sensors for chemical and biochemical sensing. Several advantages characterize photonic sensors. In particular, they exhibit high sensing performance (*i.e.*, high sensitivities and ultra-low limits of detection), low power consumption, and low cost and mass-scale production because of their CMOS-compatible technology. Moreover, the integration of photonics, microelectronics and microfluidics into the same chip represents an intriguing technological platform for the realization of lab-on-a-chip systems, which are fundamental for fast, multiplexed and real-time measurements in various application fields.

In recent years, the performance of optical biosensors became competitive with respect to other technological platforms in several applications such as security, safety, environmental monitoring, biotechnology, medical diagnostics, and food quality control [40–42].

**Figure 6.**
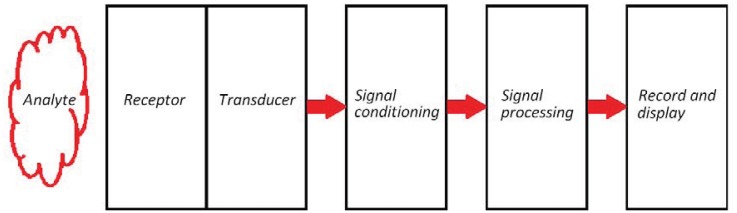
Typical operation for optical chemical sensing.

As it is sketched in the schematic of a chemical photonic sensor (Figure 6), a fundamental element characterizing optical chemical sensors is the receptor, which is generally a chemistry prerogative. In fact, it is commonly realized by depositing thin polymeric layers with nanometer thicknesses on the sensor's surface. The principal characteristic of the receptor is its high selectivity, which can be ensured because of the capability to selectively capture and immobilize a specific analyte dissolved in a complex chemical solution. Moreover, the transducer, is represented by the optical waveguide. In fact, any perturbation characterizing the surrounding medium (*i.e.*, homogeneous and surface sensing), is transduced in an effective index change of the propagating optical mode.

Integrated photonic chemical sensors are generally classified into intrinsic and extrinsic ones. In case of intrinsic sensors, the integrated optical waveguide has not only the role of guiding the optical signal along the interaction length but it also acts as a transducer. On the contrary, in the case of extrinsic sensors, there is no interaction between the chemical/biochemical species to be detected and the optical waveguide. Extrinsic devices are usually bigger and more expensive than intrinsic ones, so that they are not well suitable for mass-scale production.

**Figure 7.**
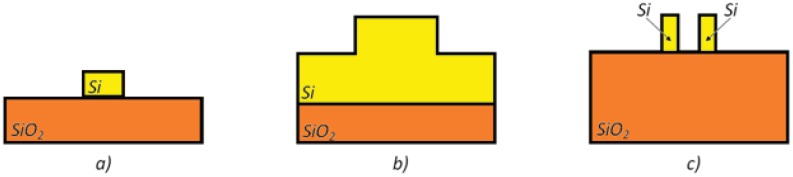
Waveguide architectures used for bio-chemical sensors: wire (**a**), rib (**b**) and slot (**c**).

Since the optical waveguide acts as a transducer in intrinsic photonic sensors, their design is crucial and strategic for ensuring high sensing performance. In case of the SOI technological platform, different types of optical waveguides have been designed for the fabrication of integrated photonic chemical sensors (see Figure 7). For rib structures, the strong confinement of the optical field in the high refractive index core results in a low light-analyte interaction in the cover medium, so that optical properties of the exposed waveguide are not strongly influenced by the concentration of chemical species to be detected. Light-analyte interaction can be improved by using wire waveguides. In fact, a wider sensor surface can be exposed to the cover medium and both interfaces between the high and low refractive index regions (*i.e.*, the top and bottom ones) experience a relevant optical transverse magnetic field.

In this context, the slot waveguide is the most suitable waveguide architecture for sensing applications. In fact, it is possible to confine a very high optical field in the slot region (*i.e.*, the low refractive index region) where the analyte to be detected is properly concentrated [43, 44]. Photonic devices based on slot waveguides enable high sensitivity and low limit of detection (LOD) (*i.e.*, the minimum resolvable optical signal corresponding to the minimum detectable analyte concentration). In case of quasi-TE polarized propagation mode, it has been demonstrated that it is possible to obtain a homogeneous sensitivity, *S_h_*, higher than 1. Consequently, an effective index change Δ*n_eff_* higher than the cover index change Δ*n_c_* can be theoretically predicted. Moreover, the dimensions of the guiding structures have been demonstrated to be relevant design parameters for the enhancement of sensing performance, as well investigated in [45].

Another important parameter to be properly selected in the design of photonic chemical sensors is the operating wavelength. For example, several gases such as carbon monoxide (CO), carbon dioxide (CO_2_), ethane (C_2_H_6_) and methane (CH_4_), to name a few, exhibit specific absorption spectra in the mid-IR region. Consequently, the propagation of optical signals at mid-IR wavelengths can significantly enhance sensing performance, because of the possibility to simultaneously employ homogeneous sensing and optical absorption. To this purpose, group IV Photonics has been recently proposed as a suitable technological platform for the realization of efficient slot waveguides for gas detection in mid-IR [46, 47]. Operating wavelengths selected for device operation are 2.883 μm and 3.39 μm. Group IV material systems and alloys (e.g., SiGeSn, GeSn, GeC, SiGeC, to name but a few) exhibit high refractive indices (*n* ∼ 4) at those mid-IR wavelengths, resulting in high refractive index contrasts and high optical field concentration in slot region. Consequently, ultra-high sensing performance has been demonstrated for homogeneous sensing of dangerous gases. In addition, such devices are characterized by relaxed fabrication tolerances and their large dimensions with respect to those characterizing conventional SOI slot waveguides, allow the propagation of second order slot modes, revealing interesting sensing performance.

### 3.1. Photonic Sensors Based on Ring Resonators

The integration of photonic waveguides in resonant microcavities, ensures a suitable optical readout at the sensor output. Ring resonators can be employed as resonant architectures for sensing applications, where the sensing principle consists in monitoring the resonance wavelength shift as a consequence of the variations of the waveguide refractive index in the presence of the substance to be detected. In particular, the resonant wavelength can be calculated as [48]:

(24) $$\lambda_{0}=\frac{2\pi rn_{\text{eff}}}{m}$$

where *r* is the ring radius of the ring, *n_eff_* is the mode effective index and *m* is an integer.

**Figure 8.**
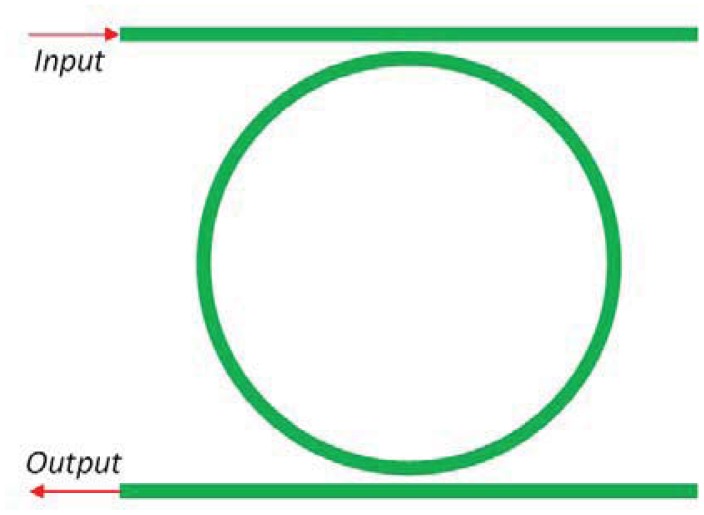
Schematic of a ring resonator with two coupled bus waveguides.

The transfer function of a single ring resonator with two bus waveguides (Figure 8) can be studied with the method based on Mason's rule and delay line signal processing [47]. This method is suitable for modeling complex structures characterized by multiple ring resonators.

The sensitivity *S_SR_* of sensors based on single ring resonators can be connected to the sensitivity of the waveguide *S_w_* (*i.e.*, homogeneous *S_h_* or surface *S_s_* sensitivities) as:

(25) $$S_{\text{SR}}={\text{FSR}}_{s}\frac{S_{w}\lambda }{n_{g}}$$

where *FSR_s_* is the free spectral range (FSR) of the sensing cavity (*i.e.*, the period of the resonant spectrum) and *n_g_* is the group index.

Integrated optical resonant microcavities have been widely proposed for the realization of photonic chemical sensors with high sensitivity and low detection limits. In particular, resonant microcavities characterized by high quality factors *Q* and low level of optical noise, present some unique advantages with respect to other technological platforms analyzed previously [49]. In particular, in resonant microcavities the interaction length does not correspond to the physical length of the device. Consequently, light propagates in multiple roundtrips into the resonant cavity (e.g., ring resonator, racetrack resonator), resulting in an overall device path length, one order of magnitude greater than the physical length characterizing the same device, as large as the quality factor (typically of the order of ∼10^6^). The typical structure of optical microcavities includes a straight waveguide coupled to a ring or racetrack resonator. Resonance wavelengths are determined as a function of the modal effective index of the optical mode propagating into the cavity. Consequently, by selecting the operative wavelength as one of the resonant wavelengths characterizing the ring resonator spectrum, variations of optical properties of the cover medium will induce an operating resonant wavelength shift Δ*λ*. The sensitivity depends on the cavity length, so that spiral structures have been proposed in order to improve sensing performances. Recently, ultra high performance have been demonstrated by using ring resonators based on slot waveguides. In particular, a sensitivity up to 2,000 nm/RIU and a LOD of 3.8 × 10^−5^ have been demonstrated.

Moreover, detection of 1.6 pg/mm^2^ in surface mass density and small molecule detection using biotin down to 10 nmol L^−1^, have been experimentally demonstrated [50]. Detailed analysis of DNA on the ring resonator surface have been achieved and interesting performance have been demonstrated such as a limit of detection of a DNA fragment of 25 bases (25-mer) (<10 pmol/L), and the detection of 150 fmol miRNA by using single-straned DNA capture probes with the capability of discriminating between single nucleotide polymorphisms. Silicon photonic microring resonators have been also used for microRNA detection [51]. In particular, authors have presented a method for the sensitive detection of microRNAs utilizing an antibody that specially recognizes DNA:RNA heteroduplexers. The intrinsic sensitivity of the microring platform coupled with the amplification provided by the anti-DNA:RNA antibodies, allows for the detection of microRNAs at concentrations as low as 10 pM. Detection of proteins such as cancer biomarkers CA15-3, HER2/ECD, CEA, TNF-α within their respective clinically significant range (*i.e.*, 1–200 ng/mL) have been efficiently detected in serum [50, 52, 53].

For further improvements of resonant microcavities performance, multiple resonant architectures have been proposed for exciting the well-known Fano resonance. In this way, it is possible to reduce the minimum detectable wavelength shift with respect to the conventional Lorentzian line-shape characterizing the transmission peaks of single-cavity resonance spectra. A limit of detection as low as 10^−8^ RIU, has been achieved with a cavity length equal to 10 mm [54].

The Vernier effect can be also excited by using multiple resonant structures, such as two cascaded ring resonators. In case of NaCl detection, a cascaded architecture has been employed for realizing a sensor operating in the wavelength range extended from 1.52 μm to 1.54 μm. This device shows a sensitivity of 2,169 nm/RIU and a LOD of 8.3 × 10^−6^ RIU, with a minimum detectable wavelength shift of 18 pm [55]. Optimal designs of chemical sensors based on Vernier effect have been performed [47], for gas detection in mid-IR [56], exhibiting homogeneous sensitivity higher than one (*S_h_* > 1) for ethane and methane detection at the operative wavelength λ = 3.39 μm.

**Figure 9.**
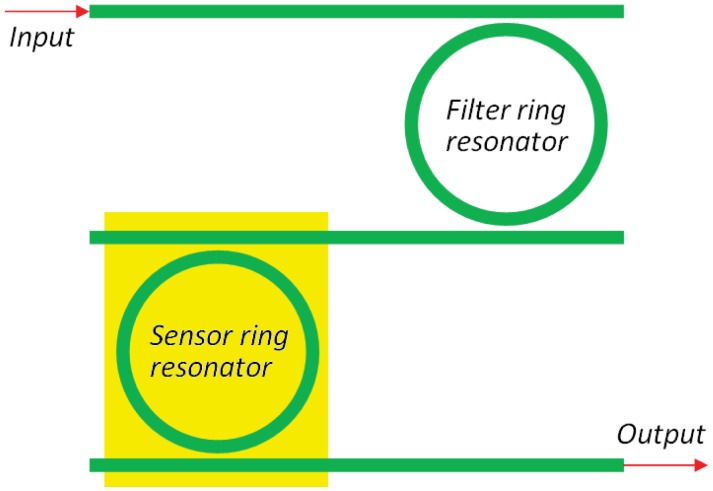
Schematic of double ring resonator architecture for excitation of Vernier effect in sensing applications.

A relevant improvement of chemical sensor based on resonant cavities is the excitation of the Vernier effect, which significantly modifies the shape and the condition for resonance [47, 55, 57–59]. In fact, by considering two cascaded ring resonators (Figure 9), it is possible to expand the overall structure FSR to the least common multiple of the FSRs of each single ring resonator.

Photonic sensors based on such an effect are usually made by two cascaded ring resonators. The former, referred to be the resonator acting as a filter, is covered by a proper cladding medium, while the latter, *i.e.*, the resonator acting as a sensor, is exposed to the analyte. The overall transmittance of the cascaded resonators can be simply calculated as the product of the two ring resonators transmittances.

It is worth noting that, if the FSR difference between the two FSRs of the two RRs is smaller than the smallest full width at half maximum (FWHM) characterizing filter and sensor RRs, the Vernier effect takes place, strongly enhancing the overall wavelength shift due to the presence of the analyte. The sensitivity *S_DR_* is strongly influenced by the excitation of the Vernier effect, so that:

(26) $$S_{\text{DR}}=\frac{{\text{FSR}}_{s}{\text{FSR}}_{F}}{\left|{\text{FSR}}_{s}-{\text{FSR}}_{F}\right|}\frac{S_{w}\lambda }{n_{g}}$$

where *FSR_F_* is the free spectral range of the filtering ring resonator [47].

### 3.2. Photonic Sensors Based on Mach-Zehnder Interferometer

**Figure 10.**
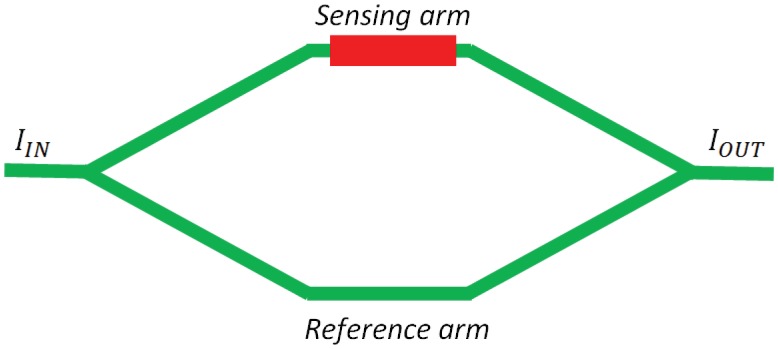
Schematic of Mach-Zehnder interferometer.

Mach-Zehnder interferometers (MZIs) have been widely proposed for chemical and biochemical sensing applications. In particular, the schematic sketched in Figure 10 represents a MZI-based architecture characterized by a sensing arm (*i.e.*, the waveguide exposed to cover medium) and a reference arm (*i.e.*, the waveguide covered by an insulating layer such as SU-8, Teflon, SiO_2_), jointed together by input and output Y-junctions. The relation between optical intensity at the sensor output and that propagating at the input section of the overall device is given as [60]:

(27) $$I_{\text{OUT}}=I_{\text{IN}}\cos^{2}\left(\frac{\Delta \varphi }{2}\right)$$

In Equation (27), $\Delta \varphi =\frac{2\pi \Delta n_{\text{eff}}L}{\lambda }$ is the phase shift due to an effective index variation Δ*n_eff_* occurring in the sensing arm characterized by a length *L*, while *I_OUT_* and *I_IN_* are output and input intensities, respectively.

Equation (27) suggests that high performances can be obtained in case of MZI sensor intensity interrogation for mm-long arms, but in this case the sensor overall dimension is not suitable for an efficient integration. For this reason, sensing and reference arms characterized by spiral paths have been proposed in order to concentrate *mm*-long arms in circular structures with a diameter of the order of few hundreds of micrometers. For example, a sensitivity as high as 4,930 rad/RIU as been experimentally demonstrated for an interferometer realized in SOI technology, with a refractive index change of 8.7 × 10^−7^ RIU/ppm of a polydimethylsiloxane (PDMS) layer deposited for sensing of benzene, toluene, ethylbenzene and xylenes [61].

However, an intensity interrogation scheme is intrinsically affected by noise, which could be a relevant problem in commercial applications. A very promising solution to this problem could be provided by a wavelength interrogation scheme. In fact, according to Equation (24), the maxima of the transmittance spectrum of a MZI experience a wavelength shift depending on the sensing arm effective index, *i.e.*, analyte concentration. The sensing principle is exactly the same as that commonly adopted for RR-based sensors, with a enhanced sensitivity intrinsically lead by the MZI architecture.

A further sensitivity improvement can be achieved by adopting the well-known Vernier effect besides the MZI wavelength interrogation. In this case, the overall sensitivity can be written as:

(28) $$S_{\text{DR}}=\frac{{\text{FSR}}_{s}{\text{FSR}}_{F}}{\left|{\text{FSR}}_{s}-{\text{FSR}}_{F}\right|}\frac{S_{w}\lambda }{\Delta n_{g}}$$

where Δ*_ng_* is the variation of the group index.

A MZI biosensor based on silicon nitride slot waveguides has been recently proposed [62]. The sensor exposed to air cladding, exhibits measured surface sensitivity *S_s_* and detection limit of 7.16 nm/(ng mm^−2^) and 1.3 (pg mm^−2^), respectively. In case of water cladding, the measured bulk sensitivity and detection limit reach 1,730(2π)/RIU and 1.29 × 10^−5^ RIU, respectively. A measured surface limit of detection of 0.155 (pg mm^−2^) has been proved by utilizing the Vernier effect through cascaded MZI architectures.

### 3.3. Photonic Sensors Based on Surface Plasmon Resonance

Another technology widely employed in photonic chemical and biochemical sensing is SPR. In particular, surface plasmons can be exploited for sensing applications since the presence of analyte located at the metal-dielectric interface causes a shift in the reflectance dip as a consequence of the change in the local refractive index. Photonic sensors based on SPR have been realized for detection of DNA, RNA, allergens and human-blood group, exhibiting a refractive index resolution as low as 1.4 × 10^−7^ [63]. In case of infrared spectroscopy, an immunosensor characterized by a sensitivity as high as 3,022 nm/RIU and a LOD of 70 pg/mm^2^ has been demonstrated by Di Pippo *et al.* [64]. Recently, a high-resolution biosensor based on localized surface plasmon resonance (LSPR) excited on an array of gold nanorods have been proposed for detection of DNA hybridization [65]. A novel approach based on the imaging of surface plasmons in polarization contrast takes advantage of the change in the polarization of light coupled to LSP on a gold nanorod array. The sensor is able to detect only one short DNA molecule per nanoparticle on average (*i.e.*, LOD ∼100 pM and surface density resolution of 35 fg/mm^2^) and measuring concentrations of short oligonucleotides down to 200 pM.

Although SPR-based sensors can exhibit high sensitivities and ultra-low LODs, sensor resolution is significantly reduced because of the impossibility to distinguish between surface refractive index change and bulk solution refractive index change, even if this drawback can be overcome by the excitation of both long range and short range surface plasmons using two dielectric layers sandwiching the metal layer [66].

### 3.4. Photonic Crystal-Based Biochemical Sensors

Photonic crystals (PhCs) have been also proposed for the realization of photonic integrated chemical sensors, with the possibility of modifying the periodic properties of the structure through the refractive index change, due to the presence of the substance to be detected [67–69].

Moreover, in PhC-based sensors it is possible to control the group velocity of the propagating optical pulse. In particular, the enhancement of the energy density of the electromagnetic field within the sensor structure can be achieved by slowing light pulse propagation (*i.e.*, the derivative of the angular frequency *ω* with respect to the wavenumber *k* approaches zero). In this way, the overlap between the optical signal and the analyte to be sensed can be strongly enhanced. Photonic sensors based on Bragg gratings can be considered as a particular photonic crystal structure [*i.e.*, 1-dimension (1D) PhC] and their principal advantage is connected to their selective range of propagating wavelengths. An optical chemical sensor in SOI technology has been proposed in [70]. It is possible to exploit two different sensing mechanisms in photonic sensors based on Bragg gratings. Firstly, the central operating wavelength shift characterizing the Bragg spectrum can be monitored as a function of the effective index change of the optical mode propagating into the guiding structure. This sensing principle allows sensitivities as high as 120 nm/RIU, useful for the detection of refractive index change of water solution [71]. Secondly, it is possible to monitor changes of the angle that the propagating light forms with the Bragg grating surface due to the presence of chemical analyte in cover medium, demonstrating a refractive index LOD as low as 1.5 × 10^−6^ RIU [72].

Nowadays, 2-D periodic structures have been considered for realization of photonic sensors, allowing sensitivity as high as 510 nm/RIU and detection limit lower than 1 × 10^−5^ RIU [73].

A two-dimensional photonic crystal microcavity has been fabricated on a SOI wafer and experimentally tested at the operative wavelength *λ* = 1.58 μm [74]. The sensor can detect dehydrated protein ad-layer thickness as low as 1 Å, revealing low selectivity because of non-specific glutaraldehyde-BSA (bovine serum albumin) binding process. In particular, the device selectivity has been demonstrated by functionalizing the sensor surface with the well-known biotin as probe molecule because of its extremely high binding affinity for streptavidin (*i.e.*, target molecule). A limit of detection as low as 2 fg of analyte has been experimentally achieved on specific biotin-streptavidin model, revealing ultra-high performance.

In conclusion, a silicon photonic wire evanescent field sensor has been fabricated on SOI wafers and exhibits a resonant shift of 1 nm when a monolayer of streptavidin protein is adsorbed to the sensor surface [75]. The grating (*i.e.*, 1 D PhC) wavelength response is estimated to be ∼1.6 pm pg^−1^ mm^2^, being the surface mass density of streptavidin monolayer of 1.6 ng mm^−2^.

### 3.5. Photonic Sensors Based on Directional Couplers

**Figure 11.**
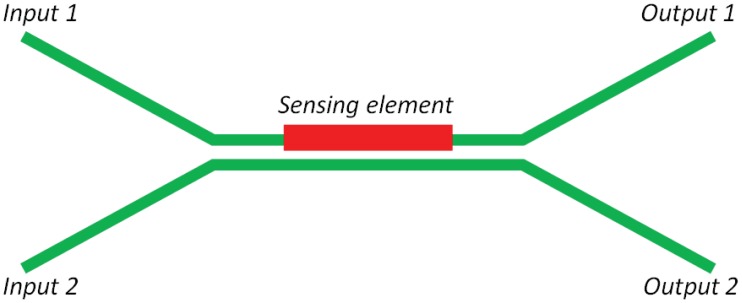
Schematic of directional coupler for sensing applications.

Integrated optical directional couplers, whose structure is shown in Figure 11, have been presented as a technological platform for chemical photonic sensors. In particular, the sensing principle is based on the power modulation at the output ports performed by the phase shift between the optical waves propagating into the two waveguides with respect to the synchronous condition. In particular, when optical propagation constants *β_1_* and *β_2_* characterizing optical modes propagating in the upper and lower arms, respectively, are equal, then the synchronous condition is verified and the coupling mechanism is ensured with the maximum coupling efficiency. The presence of any chemical analyte or gas in the coupling region can significantly compromise the synchronous condition, resulting in a minimum coupling efficiency. In this context, the architecture sketched in Figure 11 with a coupler length of 400 μm [76], can exhibit a dimensionless sensitivity as large as *S_p,i_* = 215.29, where *S_p,i_* is defined as the ratio Δ*P_i_*/Δ*n_C_* where Δ*P_i_* is the change of the normalized optical power coming out i-th output, induced by the cover medium refractive index change, Δ*n_C_*. Multichannel directional couplers have been also investigated and exploited for photonic chemical sensors. In particular, the sensing principle consists in the coupling coefficient change as a function of the cover refractive index change, indicated with Δ*k* and Δ*n_clad_*, respectively [77]. Noticeable performances have been obtained in case of a directional coupler characterized by a total length *L* = 1,607 μm. As it can be noticed from previous examples, the main drawback of these sensors is represented by their millimeter lengths, not suitable for the fabrication of photonic devices characterized by very small footprints. In particular, the analysis is focused on sensor sensitivities, detection limits, sizes and chemical analytes to be detected.

Finally, an useful comparison among different sensor configurations is reported in Table 1.

In summary, the SOI technological platform can be employed for the fabrication and mass-scale production of integrated photonic biosensors. In particular, low cost fabrication is ensured by the use of standard facilities and processes employed in microelectronics for many years now. From a technological point of view, PhC sensors can exhibit high performance with the drawback of exhibiting high sensibility as a function of geometrical parameters (e.g., hole sizes). In addition, the PhC-based sensor optical properties (*i.e.*, the PhC photonic bandgap) strongly depend on chemical/biochemical samples concentrated on the sensor surface. To this purpose, accurate design and simulations are needed for the fabrication of reliable integrated PhC sensors and to avoid undesired sensor operation. Interferometric configurations such as MZI-based sensors reveal interesting performance, are CMOS-compatible and can be produced by low-cost fabrication processes. However, ultra high performance often require long sensor architecture. SPR biochemical sensors exhibit high sensing performance (e.g., LOD ∼ pg/mm^2^) and can be fabricated in CMOS-compatible technology. However, one of the main drawbacks consists in the impossibility of detecting large target molecules like cells and bacteria because of the limited penetration of the surface plasmon evanescent field in the sensing layer (*i.e.*, ∼100 nm). In addition, properties of the metal layer employed for the excitation of localized surface Plasmon can be altered by the presence of complex chemical/biochemical samples, resulting in undesired sensor operation. In conclusion, the integration on resonant microcavities (*i.e.*, ring resonator, cascaded multiple ring resonator architectures) can exhibit ultra-high performance in biochemical sensing (*i.e.*, homogeneous and surface sensing) as it is possible to see in Table 1 (e.g., *S_SR_* = 2,169 nm/RIU and LOD ∼ 10^−6^). The main drawback is actually represented by the high refractive index contrast characterizing the air/silicon/oxide material system of the SOI technological platform. In this way, silicon photonic waveguides are sensible to any kind of imperfections (e.g., surface roughness of lateral sidewalls, sites of scattering), resulting in resonant wavelength shifts in integrated architectures based on ring resonator and, consequently, in undesired sensor operation. In this context, research efforts are actually oriented to investigate technological strategies for fabricating silicon devices with a reduced number of imperfections and low propagations losses for achieving ultra-high performance in biochemical sensing.

## 4. Photonic Sensors for Angular Velocity Measurements

Nowadays, active and fiber optical gyroscopes are widely applied in inertial navigation systems, but the mass production of a gyro with lower price, lower volume and higher reliability is fundamental for the civil market, in application fields for automotive, robots and microsatellites. The characteristics mentioned before can be achieved through the realization of integrated optical gyros (IOGs).

The principle of operation characterizing IOG is the Sagnac effect. Integrated optical gyros can be fabricated by using planar light-wave circuit (PLC) technology and photo-electronic hybrid integration technology. These technologies allow the realization of a sensitive loop with lower dimensions and the integration of active integrated devices, such as super luminescent diodes and photo detectors, on the same chip. Hence IOGs can provide compactness, stability, reliability and inexpensiveness. However, although different configurations of IOGs have been proposed in recent years, these devices are not commercially available up to now.

IOGs are typically classified into three categories, depending on whether the sensitive loop presents gain or not: active, quasi-active and passive. Active gyroscopes include a loop composed by a ring laser, which is electrically pumped. Quasi-active gyros include an optical amplifier, typically realized with ErNb-doped waveguides, which partially compensates the optical losses of the loop. The gyroscope is passive when there is no gain in the sensitive loop [80].

**Figure 12.**
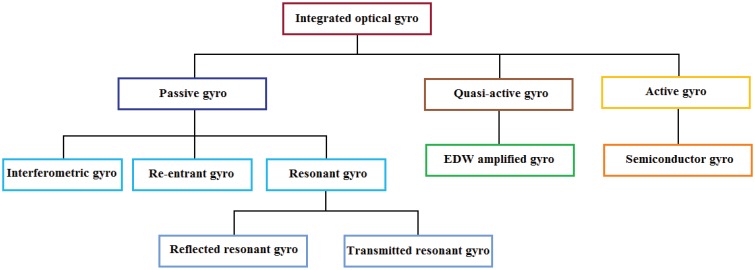
A classification of IOG configurations [81].

As fiber optic gyros, passive configurations can be further classified into interferometric integrated optical gyros (IIOGs), resonant integrated optical gyros and resonant-interferometric integrated optical gyros (RIIOGs) (also is called re-entrant gyro). The operating conditions of a passive resonant gyro are reflection mode (RRIOG) and transmission mode (TRIOG). The classification of IOGs is summarized in Figure 12.

### 4.1. Integrated Active Optical Gyros

The fundamental component of an active integrated optic gyroscope is the integrated ring laser. Starting from the first realization of the first electrically pumped integrated semiconductor ring laser (SRL), more than three decades ago, various configurations and materials have been proposed for the production of integrated ring lasers, both electrically and optically pumped.

The most relevant limiting factor of IOGs based on an active ring laser are lock-in and mode competition effects [7]. Lock-in effect is caused by the backscattering due to the roughness of the ring sidewall and leads to a frequency difference equal to zero when the angular velocity is very low, limiting the minimum detectable angular velocity. The minimum value Ω_lock_ can be reduced by increasing the cavity length. Moreover, the lock-in effect can be decreased through dithering, which can be realized introducing a constant-frequency or alternating-frequency bias between the two counter-propagating beams. Mode competition arises from nonlinear effects generated in the gain medium. It has been experimentally and theoretically demonstrated that operation in the unidirectional regime allows a narrower bandwidth than a bidirectional regime. SRLs operating in bi-CW regime in an active integrated gyro lead to the generation of optical waves with a limited power (because SRL can switch from bi-CW regime to unidirectional one by increasing the driving current) and a larger bandwidth with respect to the unidirectional regime.

An alternative operating regime of the SRL has been proposed by Sorel *et al.* [82]. This operating regime, called bi-AO, is based on the excitation of two counter-propagating resonant modes modulated by harmonic sinusoidal oscillations, with a modulation frequency typically around 100 MHz. SRL operating in this regime can allow a minimum achievable angular rate about 94 deg/h (degrees per hour) in case of quantum limited performance (*η* = 0.6, *τ* = 1 *s*).

The device includes two 1-mm-radius ring lasers coupled to a straight output waveguide. The coupling factor between the laser cavity and the waveguide is between 1% and 5% with a gap equal to 1 μm. Different values of the coupling factor are connected to the etching depth of the waveguide. At the end of the straight output waveguide two separate contacts are realized, acting as integrated photo detectors. Each contact is 50 μm long, so that the section of the waveguide which is unpumped is 50 μm long. The back reflection is reduced by applying a reverse bias to the two contacts and realizing an output waveguide tilted at 5 deg to the cleaved faces of the substrate. The total light intensity within the ring is monitored through an in-line photo detector fabricated by defining a separately biased section along the ring, ranging from 50 to 200 μm long, with a 10 μm separation to the main ring biasing contact.

Another configuration, proposed by Cao *et al.* [83], is composed by two SRLs, operating unidirectionally in order to realize an integrated optic gyroscope able to avoid the lock-in problem and decrease the counter-propagating signal linewidth. With the realization of a structure that uses both double quantum well (DQW) or quantum dot (QD) active regions, the theoretical value of the sensitivity is about 300 deg/h in case of quantum limited performance (*η* = 0.6, *τ* = 1 *s*).

**Figure 13.**
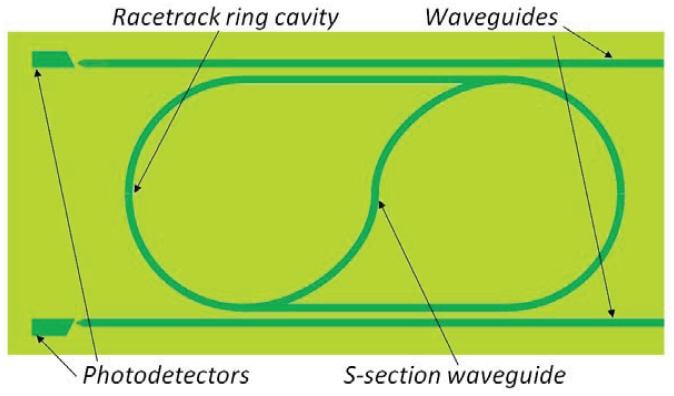
Schematic illustration of diode ring laser structure [83].

Lasers are realized with InGaAs–GaAs–AlGaAs double-quantum-well graded-index separate-confinement heterostructures, while the ring cavity is a racetrack-shaped ridge-waveguide. The ridge width is 3 μm, allowing a single-lateral-mode operation at the lasing wavelength of ∼1.02 μm. The ring radius is 1 mm and the two straight sections are 2 *mm* long, with a total cavity length of 10.28 mm. Unidirectional operation is allowed by a 3 μm wide S-shaped waveguide which connects the opposite straight sections of the ring. The S-section can be independently biased from the ring laser with a separate electrode. Coupling ridge waveguides are 3 μm wide and located 2μm away from each of the straight sections of the ring cavity, in order to realize a weak evanescent out-coupling of the ring laser light about 1%–2%. At the output ends of passive waveguides, two integrated photodiodes are fabricated with the role to detect lasing threshold and power distribution between counter-propagating modes. Waveguide end sections are tapered in order to reduce backreflections. A schematic of the integrated S-section ring diode laser is reported in Figure 13, where widths of integrated ridge-waveguide elements are evidenced in order to illustrate the structure.

**Figure 14.**
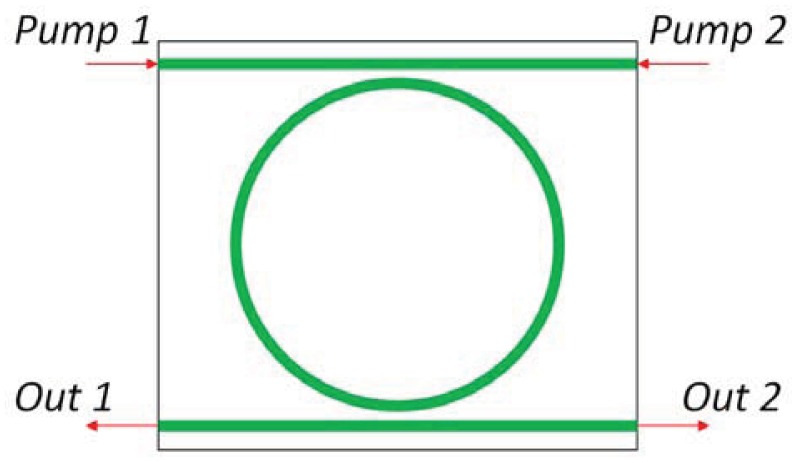
Structure of the ring laser realized with an Er-doped waveguide ring.

Optically pumped integrated ring lasers present the drawback of requiring an out-of-chip pump laser, so that they cannot be fully integrated and their efficiency is typically very low. The first optically pumped integrated ring laser has been reported in [84], realizing a cavity with a radius of 30 mm in LiNbO_3_ technology. The ring laser, whose structure is reported in Figure 14, includes an Er-doped ring with a radius of 30 mm and two straight waveguides that constitute two directional couplers. The upper waveguide couples both clockwise and counter-clockwise waves into the ring and it is optimized for operation with a TE-polarized pump wave at *λ* = 1.48 μm.

The other waveguide serves as laser output coupler, allowing to observe the guided spontaneous fluorescence and the laser emission propagating in both directions, when threshold is surpassed. If wavelength selective devices are not included into the cavity, laser emits at the wavelength of 1, 603 in TM-polarization. With a coupling efficiency of 25% at the pump wavelength, the laser threshold is about 17.5 mw coupled into the ring. Optical pumping compensates absorption and scattering losses, enhancing the resonator finesse. An estimation of absorption at the pump wavelength can be obtained from the green up-conversion light excited by a three step excitation of the Er-ions.

**Figure 15.**
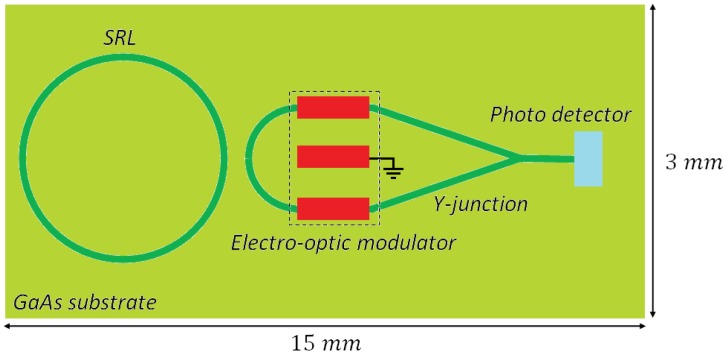
Active integrated optical gyroscope realized on a GaAs substrate.

The first optical gyroscope completely realized in an OptoElectronic Integrated Circuit (OEIC) has been theoretically proposed and patented in 2001 [85]. The structure of the optical gyro (Figure 15), which can be integrated on a 15 ×3 mm^2^ GaAs substrate, includes an AlGaAs-GaAs (DQW) circular SRL with a radius of 1.5 mm, a circular directional coupler, an electro-optic phase modulator, a Y-junction and a photo detector.

The ring laser generates two counter-propagating quasi-TE polarized optical signals, which are extracted by the directional coupler and then experience a phase shift of *π*/2. Successively, the two signals are combined in the Y-junction and, then, the photo detector generates the output photocurrent containing information about the angular rate of the device. Polarization selectivity obtained through an optimal design of the gain medium is fundamental for achieving an optical gain for quasi-TM polarization much lower than for quasi-TE, in order to reduce the noise related to coupling between the two polarizations.

**Figure 16.**
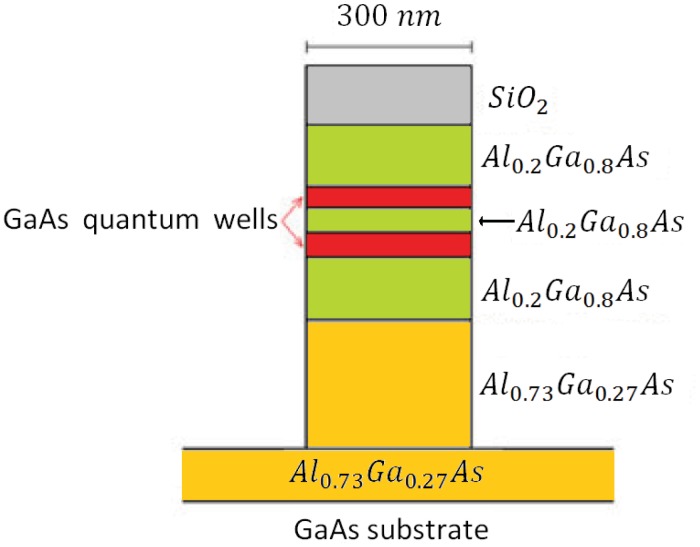
Optical passive waveguide cross-section of the GaAs integrated optical gyro sensor.

The optical waveguide in the SRL includes an Al_0.2_Ga_0.8_As barrier which separates the two GaAs quantum wells, two cladding layers to be realized in Al_0.2_Ga_0.8_As and a Al_0.73_Ga_0.27_As buffer. Waveguide cross-section is shown in Figure 16. When the sensor rotates the two counter propagating beams exhibit a frequency difference proportional to the rotation rate, as a consequence of Sagnac effect. When the two waves interfere in the Y-junction, the amplitude of the output wave oscillates with a frequency proportional to the rotation-induced frequency difference, so that the device angular rate can be estimated by measuring the frequency of electrical signal generated by the photodiode.

As all active optical gyros, this device suffers from lock-in effect, so that angular rates inferior to 210 deg/h cannot be estimated since the counter-propagating beams do not experience any frequency difference. In the lock-in operating region, only the phase rotation-induced phase shift can be used to measure the angular rate. However, the phase shift depends not only on the angular rate, but also on many SRL technological parameters, such as radius and backscattering coefficient, so that the sensor accuracy depends on these technological parameters. The sensor resolution in the lock-in operating region is improved by the shift introduced by the electro-optic modulator. In lossless condition, minimum detectable angular rate of this integrated gyro has been estimated of about 0.01 deg/h.

**Figure 17.**
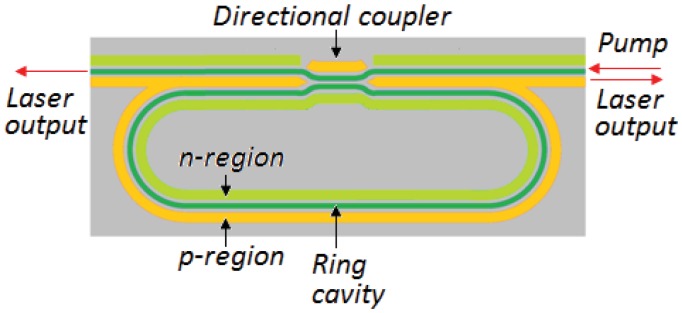
Layout of the silicon ring laser cavity with a p-i-n structure along the waveguides.

Very recently, another large cavity length (30 mm) optically pumped integrated ring laser has been fabricated in Silicon-on-Insulator technology, employing the Raman effect [86]. This device (lasing at 1,686 nm) exhibits single longitudinal mode operation and a very reduced linewidth equal to 80 kHz. The monolithic integrated cavity is constructed from a low-loss silicon-on-insulator (SOI) rib waveguide forming a race-track shaped ring resonator on a single chip (Figure 17). A bus waveguide is connected with the ring cavity via a directional coupler which couples both pump and signal laser light into and out of the cavity. The coupling ratio depends on input wavelength and polarization and can be varied by changing the gap and/or length of the coupler.

**Figure 18.**
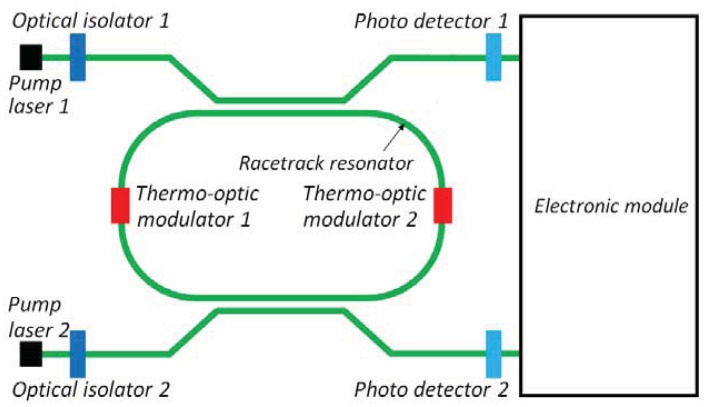
Layout of the Raman integrated optical gyroscope in SOI technology.

The SRL investigated in [86] has been proposed for the realization of an active integrated optical gyro by Passaro *et al.* [7]. The device scheme is reported in Figure 18. Two pump waves at *λ* = 1,433 nm are coupled into the racetrack resonator through two directional couplers. Stokes waves at *λ* = 1,546 nm are generated into the cavity through Raman effect and extracted in order to detect the angular rate through electronic elaboration of the photocurrents generated by the photo detectors at the output of the bus waveguides.

Both racetrack and bus waveguides are realized with a rib waveguide in SOI technology. The total length of the cavity is 3 cm, while the radius is equal to 400 μm. The coupling factors are 30% and 15% at pump and Stokes wavelengths, respectively. These parameters are fundamental for the performance of the optical gyro, since they are connected to the pump power threshold for the Raman effect excitation. Optimal performance of the laser can be obtained with a critical coupling for the pump wave (losses are equal to gain) and low coupling factor at the Stokes wavelength. The pump power threshold is calculated to be equal to 93 mW, while for a pump power of 600 mW the output Stokes power is 177 mW. The sensitivity is 0.082 deg/s in the case of a pump power of 600 mW. The two thermo-optic modulators are included to perform optical dithering and to avoid lock-in effect. The maximum detectable angular velocity of this device is as low as 101 rad/s.

### 4.2. Passive Integrated Optical Gyros

Ring resonators with dimensions of the order of millimeters are widely applied for the realization of optical integrated passive gyroscopes. The operation of the angular rate sensor is based on the excitation of two propagating waves into the resonator, clockwise and counterclockwise. Passive IOGs usually include an optical cavity coupled to one or two bus waveguides, as in the case of active IOGs, but they do not present any element able to introduce gain in the system. In case of a single bus waveguide, the two ends can be used as input and output ports. On the contrary, when two bus waveguides are included into the sensor, through or drop configuration can be exploited, since output waves can be extracted from the same waveguide where input waves are launched in or from the other one. A critical aspect is represented by the same power required to counter-propagating waves, since a difference should result in an unpredictable bias in the sensor output.

Optical losses are the most critical feature of guiding structure used in integrated IOGs, since they influence the cavity quality factor. Usually, a cavity quality factor *Q* > 10^5^ is needed to obtain a good performance of the device. An appropriate value of this parameter can be obtained by using low-loss waveguides or including active elements in the system. Low loss waveguides with absorption coefficients lower than 0.1 dB/cm at 1.55 μm have been fabricated in Silica-on-Silicon (SOS) technology, allowing the realization of ring resonator with high quality factors. The highest experimental quality factor in SOS technology, equal to 2.4 × 10^7^, has been measured in case of a 5 μm × 5 μm phosphorus-doped core with a boron-and-phosphorus-doped glass top cladding. The index contrast at the operating wavelength of 1.55 μm is 0.7% and the absorption coefficient is about 0.01 dB/cm [87]. In this context, recent research efforts are oriented to the design and fabrication of ultra low loss waveguides on silicon [88, 89] and silicon nitrate (Si_3_N_4_) based platforms [90]. The relevance of optical losses in passive IOGs has been recently demonstrated in [91], where an analytical model has been developed in order to allow an optimal design of the device. Besides the waveguide absorption coefficient, other key parameters, such as cavity length, coupling factors and detuning factor, have been included in the model of IOGs.

**Figure 19.**
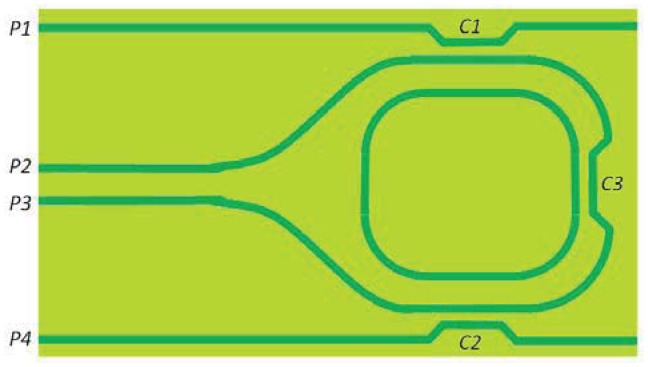
Layout of the integrated optical gyroscope in SOS technology.

Sensing performance can be further improved including active elements in the system (as an semiconductor optical amplifier, SOA), realizing quasi-active integrated optical gyroscopes. Silica-on-Silicon technology has been recently employed for the demonstration of an integrated optical gyroscope by Huai-Yong *et al.* [92]. The structure of the sensor, sketched in Figure 19, includes three couplers and some straight and curved waveguides. Clockwise and counterclockwise input waves are injected into ports *P2* and *P3*, and coupled out from ports *P4* and *P1*, respectively. The scheme used for testing the device includes a laser emitting at *λ* = 1,550 nm with a linewidth of 30 kHz and two detectors with responsivity of 0.95 A/W. The fundamental detection limit of this IOGs is calculated to be 1.6 deg/h, while cavity finesse is 70.

The main sources of losses are the splitting rates of the couplers and the propagation loss coefficient. The former contribution can be controlled with an appropriate design of the coupler C3, while propagation losses in SOS technology can reach values less than 0.01 dB/cm. The total transmission loss of the device is measured to be 6.8 dB. The design of the device should also consider the realization of a single-mode waveguide with a modal distribution that can match the mode-field diameter of the fiber used to inject light into the integrated circuit.

An IOG based on GeO_2_-doped silica waveguides has been also proposed [93]. The configuration of the sensor is similar to that sketched in Figure 19, but the phase difference between CW and CCW beams is controlled with two acousto-optic modulators positioned before the input ports. The waveguide core is 5.4 μm × 5.4 μm wide, with refractive indices of core and cladding equal to 1.4561 and 1.4451, respectively. The coupling ratios of couplers *C1* and *C2* are equal to 50%, while in case of *C3* the coupling ratio is 30%. The laser source used in the experimental setup emits light at *λ* = 1.550 nm with a spectral linewidth of 50 kHz. The measured propagation losses are 0.02 dB/cm and 0.1 dB per round trip, while the cavity finesse is 12.5.

Performance of IOGs in silica waveguides can be significantly improved with the use of the Single Phase Modulation Technique (SPMT) [94] or Double Phase Modulation Technique (DPMT) [95]. The integrated optical gyroscope presented in [94] is based on a 6-cm-long ring realized on a silica planar lightwave circuit (PLC) with a control system of the state of polarization of incoming light. The FSR, FWHM and finesse of the resonator are equal to 3.4 GHz, 62 MHz and 54.8, respectively. The scheme realizes SPMT through two LiNbO_3_ phase modulators inserted before the polarization controllers at the input of the PLC. The feedback circuit is used to set the emission frequency of the fiber laser at the resonance frequency of CCW wave through lock-in technique. As a consequence, the output at the photo detector connected to the CCW wave is constant, while the frequency difference due to Sagnac effect can be obtained from the frequency difference between the driving frequency of the two acoustic-optical modulators (AOM). The device sensitivity is 7.3 × 10^−5^ rad/s.

Realization of DPMT can be useful to achieve additional carrier suppression, including two phase modulators for each signal wave. This technique has been employed in a scheme for optical gyroscopes proposed in [95]. The sensing element is a silica waveguide resonator with a total length of 7.9 cm, so that cavity FSR, FWHM and finesse are equal to 2.61 GHz, 56.3 MHz and 46.3, respectively. Differently from PLC considered in [94], this scheme employs polarization maintaining silica waveguides.

**Figure 20.**
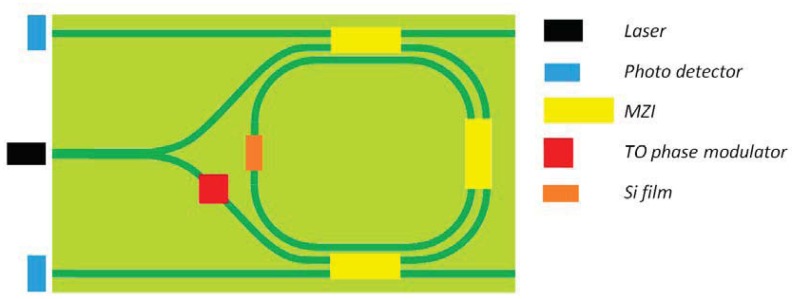
Integrated optical gyroscope on silica PLC.

The first angular rate sensor completely integrated in an optical circuit has been proposed by Suzuki *et al.* [96]. The device, whose scheme is reported in Figure 20, is realized on a silica PLC and it is based on resonator microoptic gyro (MOG). Noise sources in the sensor are connected to polarization fluctuation and backscattering. The first effect, *i.e.*, polarization fluctuation, can be reduced with an appropriate design of waveguide birefringence, while the other one can be reduced by introducing a thermo-optic (TO) phase modulator and by realizing binary phase shift keying (B-PSK) modulation. The four integrated switches based on MZI are used to alternatively lock laser frequency to CW and CCW resonance frequencies and measure the angular velocity at which the device is rotating. The gyro operates at 1,550 nm with *δΩ* ∼10 deg/h.

Recently, a dual-resonator structure for IOGs based on silica waveguides has been proposed in [97], in order to further reduce the performance limitations due to back reflection and Kerr effect. Ion exchange and Titanium in-diffusion in Lithium Niobate (LiNbO_3_) are used to realize low-loss waveguides, even if the absorption coefficient is quite larger than that in SOS technology. The main advantages of these glass waveguides are their simple and economical manufacturing process and the availability of various dopants, enabling optical amplification into the waveguide.

**Figure 21.**
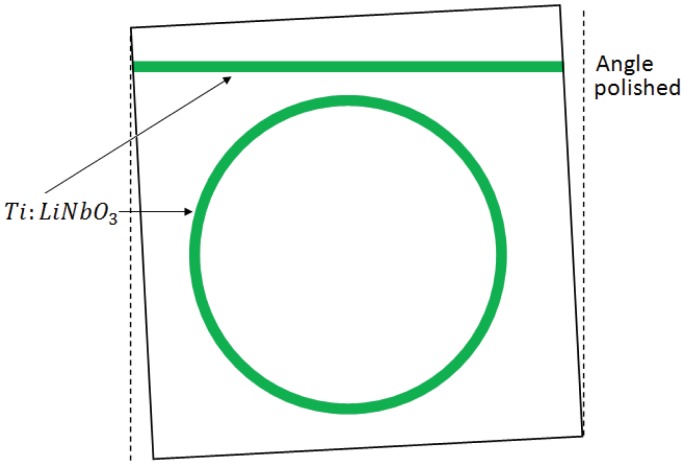
Passive integrated ring resonator realized in Ti: LiNbO_3_ technology.

Loss compensation by optical gain has been exploited in an angular rate sensor [98], allowing quality factor exceeding 10^7^. The device includes a 56.07 mm-long racetrack resonator realized with neodymium doped-silicate. The operating wavelength is 1.02 μm, while the pump wave has a power of 150 mW at the wavelength of 0.83 μm. The structure includes two bus waveguides, one for the pump wave and the other one for the signal. The total quality factor Q is about 1.89 × 10^7^, corresponding to a finesse F = 250. Design of a high finesse Ti: LiNbO_3_ integrated optical ring resonator is reported, too [99, 100]. The structure in Figure 21 consists of a single mode ring resonator with a diameter of 60 mm, operating at *λ* = 1,550 nm. The cavity is coupled to a straight waveguide, whose ends are both input/output ports.

Optical losses are measured to be about 0.03 dB/cm for TE polarization, while bending losses can be neglected for radius >25 mm, as in this case. Cavity FSR and FWHM are equal to 830 MHz and 80 MHz, respectively. The laser source emits at λ = 1,550 nm with a spectral linewidth of 150kHz. The minimum rotation rate is calculated to be 6.7 deg/h. The cavity quality factor is measured to be about 2.4 × 10^6^, corresponding to a finesse of 10.

**Figure 22.**
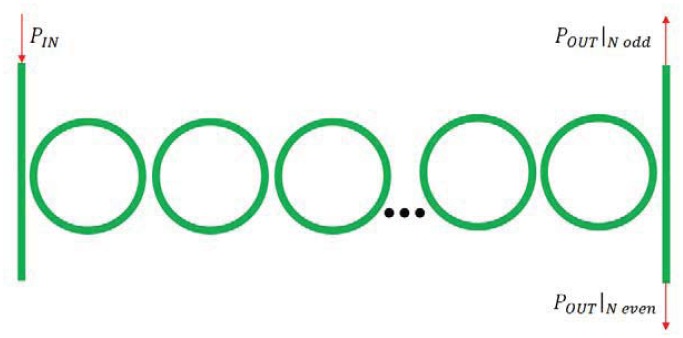
Example of CROW architecture.

Coupled-Resonator Optical Waveguide (CROW) or Side-Coupled Integrated Spaced-Sequence of Resonators (SCISSOR) have been recently proposed as a new solution for high performance IOGs. CROW structures are composed by chain of coupled resonators in which light propagates because of the coupling between adjacent resonators, while SCISSOR structures includes sequence of resonators evanescently coupled with a bus waveguide. The main advantage connected to these two structures is the possibility of slowing light by decreasing its group velocity. CROW architecture typically includes a sequence of *N* directly coupled microring resonators, as sketched in Figure 22 [38].

In particular, each ring has a physical length *L* with free spectral range *FSR* = *c*/*nL*, being *n* = 3.0 the effective refractive index in case of silicon waveguide. The coupling coefficients are denoted as *σ_i_*, where *i* indicates the coupling regions, numbered from 1 to *N* + 1. All couplers are assumed ideal, neglecting the coupling losses and backreflections, so that they can be modeled with coupling coefficients *τ* and *σ*, related by the following expression:

(29) $$\tau^{2}+\sigma^{2}=1$$

**Figure 23.**
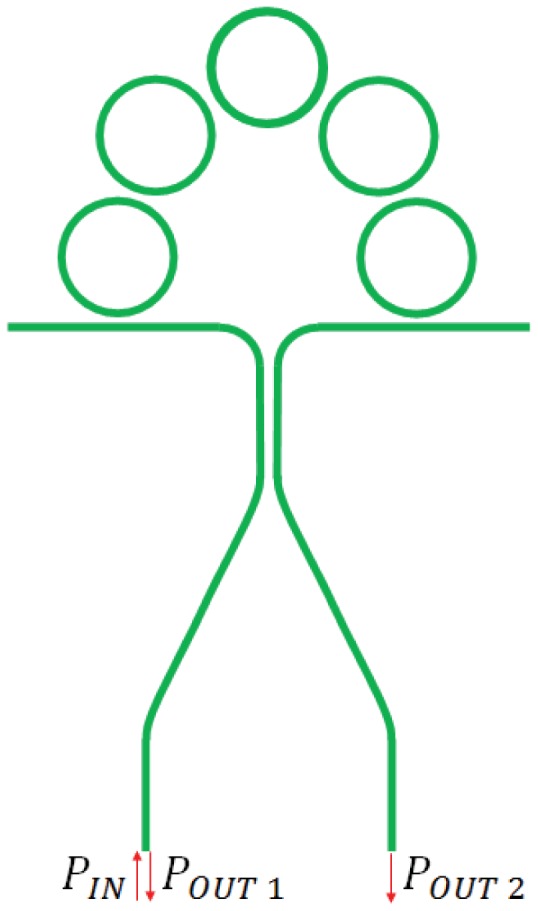
Passive integrated gyro based on CROW architecture.

A CROW-based passive integrated optical gyro has been demonstrated in [101]. The structure, reported in Figure 23, includes a 3 dB power divider which splits the input laser beam into two signals which counter propagate into the CROW architecture. When the device is motionless, the phase shift due to the propagation is identical for the two signals. If the sensor rotates, the two signal experience different phase shifts, so that they are not in phase and present phase shift *Δϕ* at the output. When the two output signals interfere at the directional coupler, the output power depends on *Δϕ* according to the following equations [76]:

(30) $$P_{\text{out},1}=P_{\text{in}}\cos^{2}\left(\frac{\Delta \phi }{2}\right)$$

(31) $$P_{\text{out},2}=P_{\text{in}}\sin^{2}\left(\frac{\Delta \phi }{2}\right)$$

where *P_in_* is the optical power of the laser beam. Through Equations (30)–(31) it is possible to estimate *Δϕ* and, consequently, the rotation rate, from the measure of optical powers at the two output ports.

**Figure 24.**
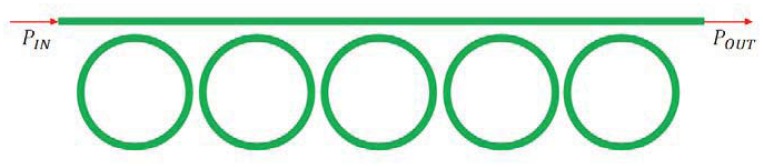
SCISSOR architecture.

The typical configuration of SCISSOR architecture is reported in Figure 24 [102]. The transfer function of each unit cell can be written as:

(32) $$H_{\text{ring}}=\frac{E_{\text{out}}}{E_{\text{in}}}=\frac{\tau -e^{j\phi }}{1-\tau e^{j\phi }}$$

where $\phi =\frac{2\pi n}{\lambda }L$. The transfer function possesses one pole (*p*_1_ = 1/*r*) dependent on its zero (*z*_1_ = *r*). If the structure is composed by *N* resonators, the total transfer function is $H_{\text{ring}}^{N}$.

The phase shift due to Sagnac effect is proportional to the group index, so it can be enhanced in SCISSOR structures as a consequence of the larger value of group index achievable through this architecture [103, 104].

## 5. Photonic Sensors for Electric Field Measurements

Electromagnetic field sensors are becoming more and more important in the field of electromagnetic compatibility (EMC) and electromagnetic interference (EMI) for various applications, such as telecommunications, military applications, materials processing, and health services, among others. The most interesting benefit of photonic sensing of electric fields is the possibility of realizing electrical isolation from electrical fields, since they are realized with dielectric materials. Moreover, technological characteristics allow low perturbation and high sensitivity, so that it is possible to preserve both phase and amplitude of the electric field.

Various technological platforms and configurations have been proposed for the realization of electric field sensors. The operation principle is usually the modulation of the optical intensity performed by the applied voltage. In turn, the voltage can be connected to the external electric field through the antenna factor. Electro-optic effect is typically the sensing principle of sensors in LiNbO_3_ technology, where the sensitivity is directly proportional to the length of the sensing region. However, device length is inversely related to the bandwidth, so that an optimal design needs a trade-off between these two conflicting requirements. Typical configurations exploit Mach-Zehnder or Fabry-Perot interferometers, but the second configuration allows higher sensitivity for the same interaction length.

Electric fields sensors in LiNbO_3_ based on Mach-Zehnder interferometers are commercially available [105, 106]. For example, the device proposed in [105] is composed of a semiconductor laser whose output is connected to the integrated circuit through an optical fiber, which is also used to transport the output signal to the photo detector. The integrated optical circuit is 1 mm × 20 mm wide and its connection and alignment to the optical fiber is improved by the realization of V-grooves on the chip. Another relevant characteristic of this sensor is the use of a retro-reflective optical modulator, which allow downsizing of the device. In [106] an asymmetric Mach-Zehnder configuration is exploited for the sensor, allowing a chip size of 6 mm × 26 mm and total sensor size of 80 mm × 10 mm ×10 mm. The minimum detectable electric field of these sensors is about 0.22 mV/m at 50 MHz, while the 3 dB bandwidth is 300 MHz. The main limitation to sensing performance is due to the fact that electrodes have to be sufficiently long in order to achieve high sensitivity, but at the same time they reduce the bandwidth under 1 GHz.

An approach adopted for improving the performance of electric field sensor based on LiNbO_3_ modulators have been proposed in [107]. The most relevant elements of this sensor is a small size coplanar waveguide antenna, which is connected directly to the z-cut LiNbO_3_ substrate, and the travelling wave electrode used in the Mach-Zehnder modulator. The half-wave voltage of the modulator is 4 V, while the minimum detectable electric field intensity is 0.89 mV/m at 2 GHz, with a linear sensor response up to 9 V/m.

Another technological improvement for high sensitivity electric field sensors in LiNbO_3_ has been proposed in [108]. In this paper, a tapered antenna array configuration is realized on a Mach-Zehnder interferometer, allowing detection of electric fields at frequencies ranging from 10 kHz to 18 GHz. In particular, the spectral response shows variations lower than 2 dB in the range 10 kHz−1 kHz and less than 10 dB in the range 1 kHz–18 kHz. The minimum detectable electric field is 0.4 V/m, with a linear behavior of the sensor up to 18.48 V/m and a maximum detectable electric field equal to 103 V/m.

Mach-Zehnder modulators have been also proposed for detection of intensive electric fields [109]. In this case, the length characterizing the two arms is 40 mm, with a distance of 60 μm, but at the same time an optical path difference is realized in order to obtain a bias of π/2 useful to reach an higher linear response. The integrated optical circuit is realized on an x-cut LiNbO_3_ substrate and it is 55 mm × 6 mm wide. The most relevant performance of this sensor is the possibility of detecting electrical field intensities above 250 kV/m. In [110] the performance of the same interferometer with three different electrode configurations has been analyzed in case of intensive electric fields detection. Experimental results show that a mono-shield electrode design is preferable for architectures with more than one metallic element, such as two electrodes connected with a vertical or horizontal dipole antenna.

Recently a new configuration of electric field sensor in LiNbO_3_ without any metallic element has been proposed [111]. The device is based on a waveguide near cut-off realized in a inverted domain region. The sensing principle is the refractive index change caused by electro-optic effect between positive and negative domains, which leads to a mode profile broadening. This situation leads to optical losses that can be measured in order to estimate the mode profile mismatch between the two regions and so the electric field. The device has been tested for operation with DC electric field up to 2.6 MV/m and RF intensities from 19 V/m to 23 kV/m.

Recently polymeric waveguides on silicon substrate have been considered for optical electric field sensors [112]. The structure is based on a Y-fed directional coupler realized with a domain inverted electro-optic polymer. Phase modulation reversal between the two arms of the directional coupler is obtained without a domain-inverted poling of the waveguide by using a poling electrode that zigzags from one waveguide to the other. The external electric field induces equal phase modulation with reversed polarity on the two arms when the poling electrode is absent. The sensor exhibits an operating range extended from 16.7 V/m to 750 kV/m and a noise free dynamic range of 70 dB.

Another sensing principle exploited for optical electric field sensor is electro-absorption. The advantages of modulators based on this optical effect over electro-optic interferometers, is represented by lower interaction lengths and efficient operation at frequencies up to 1GHz. The material employed for the fabrication of these devices is typically InP and its compound alloys with Al and As. High efficiency and large bandwidth can be obtained when quantum confined stark effect (QCSE) is excited in the structure, achieving spectral operation in the range extended from 10 GHz to 6 GHz and a minimum detectable electric field intensity of 16 mV/m [113, 114].

**Figure 25.**
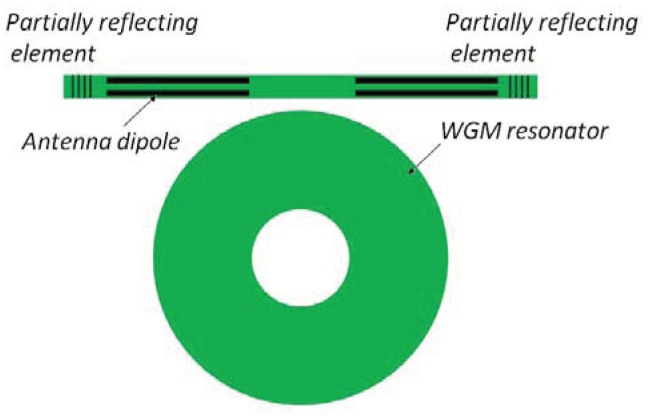
Architecture of the electric field sensor in SOI technology.

Electric field sensors in SOI technology, whose structure is reported in Figure 25, have been proposed in [11, 115]. The sensing element of the device proposed in [115] is a Whispering-Gallery Mode (WGM) resonator, which is coupled to a straight waveguide where a Fabry-Perot (FP) cavity is realized through two reflecting elements (gratings).

Coupling between the WGM resonator and FP cavity significantly modifies the transmission spectrum of the structure, since it induces an asymmetric Fano resonance response. The most relevant characteristic of this spectrum is the rapidly varying slope between the zero and the unity transmission at the resonance wavelength, which is about three or four times higher with respect to that achievable by a single disk resonator. Moreover, the resonant spectrum and its shift from the resonance depend on the effective index of the optical mode propagating into the straight waveguide. This dependence is useful since it is possible to obtain a variation of the effective mode index, and consequently of the resonance line shape, in the presence of an external applied voltage. In this architecture, the effective index variation is caused through the plasma dispersion by a metal oxide semiconductor (MOS) capacitor realized inside the FP cavity. It is important to note that sensitivity is significantly increased if the antenna dipole is included on the straight waveguide and not on the WGM resonator, which would experience higher optical losses if covered by a metalized region. Two measuring techniques can be exploited: the wavelength shift of the transmitted output optical beam (wavelength interrogation) or the output amplitude at the same wavelength (amplitude interrogation).

**Figure 26.**
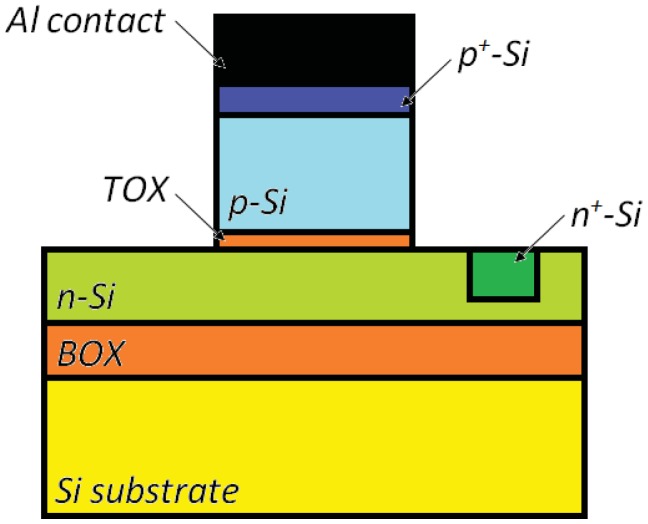
Rib waveguide with MOS structure (BOX and TOX stand for buried oxide and thin oxide, respectively).

The rib waveguide used in the sensor, whose cross section is sketched in Figure 26, supports only the quasi-TE fundamental mode and includes the MOS structure. An optimal design of waveguide dimensions is required in order to obtain a significant influence of the MOS structure without introducing high optical losses, which can be obtained if the MOS capacitor works in accumulation regime. The design of the WGM structure takes into account the reduction of birefringence between the fundamental quasi-TE and quasi-TM modes and reduce the loss contribution due to propagation, back scattering, bending and leakage to the substrate.

In the design performed in [115], the disk resonator radius and straight waveguide total length are equal to 150 μm and 1.8 mm, respectively. Moreover, the resonance wavelength is set to be 1.55 μm. It has been demonstrated that the performance of this device are superior than those exhibited by electric field sensors based on MZIs in LiNbO_3_, since the minimum detectable electric field is 0.015 mV/m, corresponding to a minimum detectable voltage of 60 mV. The operating range goes from −0.25 mV/m to 0.25 mV/m. Moreover the bandwidth of the sensor is equal to 500 MHz, which is enough for various applications of electric field sensors.

**Figure 27.**
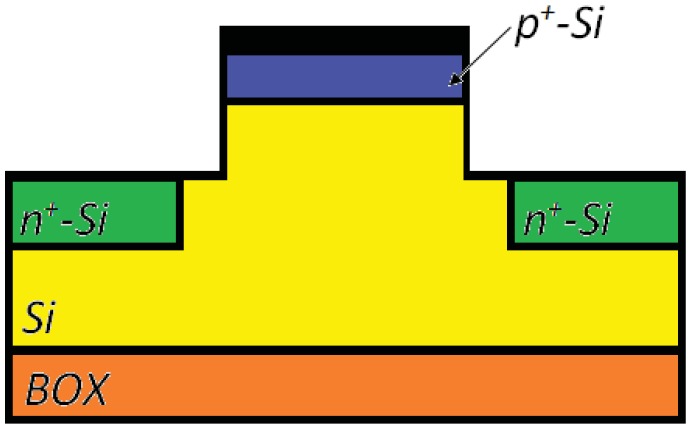
Rib waveguide with p-i-n structure.

A similar architecture is proposed in [11], but in this case the effective index variation through plasma dispersion is obtained by a p-i-n diode realized on a large rib waveguide, as can be seen in Figure 27. The realization of p-i-n structure leads to detect voltages down to 5 mV, which is significantly less than the value demonstrated in case of a MOS capacitor. Moreover, in Ref. [11] performance of the sensor in case of large rib, micro rib and sub-micron rib waveguides have been analyzed, showing that large rib structures allow detection of lower electric fields but, at the same time, lead to a lower variation of the modal effective index as a function of the voltage applied to the structure.

## 6. Conclusions

Photonic sensors are becoming more and more attractive in different application fields such as chemistry, medicine, biotechnologies, automotive, aeronautic, aerospace, to name but a few. High sensitivity, low cost, integration with CMOS electronic read out and real time processing are the most important strengths that characterize photonic sensors. Moreover, photonic sensing is a feasible and intriguing alternative to conventional sensing systems because of the development of mature technologies, in particular of the Silicon-on-Insulator platform.

The present review starts from the theoretical explanation of the operating principles of photonic sensors: homogeneous sensing, surface sensing, absorption-based sensing, Vernier effect, Stimulated Raman Scattering, Sagnac effect and Fano resonance. The state of the art of photonic sensors is presented as a function of specific application area, thus for chemical, biochemical, gyroscope and electric field measurements, focusing on the most recent technological and architectural improvements. In particular, for chemical and biochemical applications, the excitation of Vernier effect in double ring structures is fundamental to improve the sensitivity in case of wavelength interrogation. Optical integrated gyroscopes based on microcavities present the possibility of obtaining the same performance of actual optical integrated fiber gyros, but with a lower cost and better integration with electronic read out. In the case of electric field sensors, the development of devices based on interaction between two cavities and excitation of Fano resonance allow better sensitivity.

Accurate modeling of these architectures, as well as numerical simulations, allows the optimization of photonic sensors and the exploitation of all their characteristics, so that their fields of application will become greater and greater thanks to their incomparable advantages.

## Figures and Tables

**Table 1.** Comparative analysis of several silicon photonic platforms for biochemical sensing.

**Architecture**	**Technology**	**Performance**	**Size**	**Analyte**

MZI [61]	SOI	8.7 × 10^−7^ RIU/ppm	2.1 mm-long	BTEX
MZI [78]	CMOS-compatible	0.3 pg/mm^2^	1.8 mm-long (×9-array)	IgG goat, rabbit
SPR [64]	CMOS-compatible	3,022 nm/RIU 70 pg/mm^2^	∼800 μm^2^	Molecules
PhC [73]	SOI	510 nm/RIU 1 × 10^−5^ RIU	2 μm-cavity length	Gases: N_2_, He, CO_2_
Directional coupler [76]	SOI	0.1 g/L	∼1 mm^2^ (footprint)	Glucose
Ring resonator [79]	SOI	60 fM	175 × 500 μm^2^ (×32-array)	DNA
Cascaded resonators [55]	SOI	2,169 nm/RIU 8.3 × 10^−6^ RIU	200 × 70 μm^2^ (×2-array)	NaCl, molecules
